# The policies on the use of large language models in radiological journals are lacking: a meta-research study

**DOI:** 10.1186/s13244-024-01769-7

**Published:** 2024-08-01

**Authors:** Jingyu Zhong, Yue Xing, Yangfan Hu, Junjie Lu, Jiarui Yang, Guangcheng Zhang, Shiqi Mao, Haoda Chen, Qian Yin, Qingqing Cen, Run Jiang, Jingshen Chu, Yang Song, Minda Lu, Defang Ding, Xiang Ge, Huan Zhang, Weiwu Yao

**Affiliations:** 1 Laboratory of Key Technology and Materials in Minimally Invasive Spine Surgery, Tongren Hospital, Shanghai Jiao Tong University School of Medicine, Shanghai, China; 2 Center for Spinal Minimally Invasive Research, Shanghai Jiao Tong University, Shanghai, China; 3 Department of Imaging, Tongren Hospital, Shanghai Jiao Tong University School of Medicine, Shanghai, China; 4 Department of Epidemiology and Population Health, Stanford University School of Medicine, Stanford, CA USA; 5 Department of Biomedical Engineering, Boston University, Boston, MA USA; 6 Department of Orthopedics, Shanghai Sixth People’s Hospital, Shanghai Jiao Tong University School of Medicine, Shanghai, China; 7 Department of Medical Oncology, Shanghai Pulmonary Hospital, Tongji University School of Medicine, Shanghai, China; 8 Department of General Surgery, Pancreatic Disease Center, Ruijin Hospital, Shanghai Jiao Tong University School of Medicine, Shanghai, China; 9 Department of Pathology, Shanghai Sixth People’s Hospital, Shanghai Jiao Tong University School of Medicine, Shanghai, China; 10 Department of Dermatology, Shanghai Ninth People’s Hospital, Shanghai Jiao Tong University School of Medicine, Shanghai, China; 11 Department of Pharmacovigilance, Shanghai Hansoh BioMedical Co., Ltd., Shanghai, China; 12 Editorial Office of Journal of Diagnostics Concepts & Practice, Department of Science and Technology Development, Ruijin Hospital, Shanghai Jiao Tong University School of Medicine, Shanghai, China; 13 MR Scientific Marketing, Siemens Healthineers Ltd., Shanghai, China; 14 MR Application, Siemens Healthineers Ltd., Shanghai, China; 15 Department of Radiology, Ruijin Hospital, Shanghai Jiao Tong University School of Medicine, Shanghai, China

**Keywords:** Guideline, Radiology, Natural language processing, Artificial intelligence, Meta-research

## Abstract

**Objective:**

To evaluate whether and how the radiological journals present their policies on the use of large language models (LLMs), and identify the journal characteristic variables that are associated with the presence.

**Methods:**

In this meta-research study, we screened Journals from the Radiology, Nuclear Medicine and Medical Imaging Category, 2022 Journal Citation Reports, excluding journals in non-English languages and relevant documents unavailable. We assessed their LLM use policies: (1) whether the policy is present; (2) whether the policy for the authors, the reviewers, and the editors is present; and (3) whether the policy asks the author to report the usage of LLMs, the name of LLMs, the section that used LLMs, the role of LLMs, the verification of LLMs, and the potential influence of LLMs. The association between the presence of policies and journal characteristic variables was evaluated.

**Results:**

The LLM use policies were presented in 43.9% (83/189) of journals, and those for the authors, the reviewers, and the editor were presented in 43.4% (82/189), 29.6% (56/189) and 25.9% (49/189) of journals, respectively. Many journals mentioned the aspects of the usage (43.4%, 82/189), the name (34.9%, 66/189), the verification (33.3%, 63/189), and the role (31.7%, 60/189) of LLMs, while the potential influence of LLMs (4.2%, 8/189), and the section that used LLMs (1.6%, 3/189) were seldomly touched. The publisher is related to the presence of LLM use policies (*p* < 0.001).

**Conclusion:**

The presence of LLM use policies is suboptimal in radiological journals. A reporting guideline is encouraged to facilitate reporting quality and transparency.

**Critical relevance statement:**

It may facilitate the quality and transparency of the use of LLMs in scientific writing if a shared complete reporting guideline is developed by stakeholders and then endorsed by journals.

**Key Points:**

- The policies on LLM use in radiological journals are unexplored.
- Some of the radiological journals presented policies on LLM use.
- A shared complete reporting guideline for LLM use is desired.

**Graphical Abstract:**

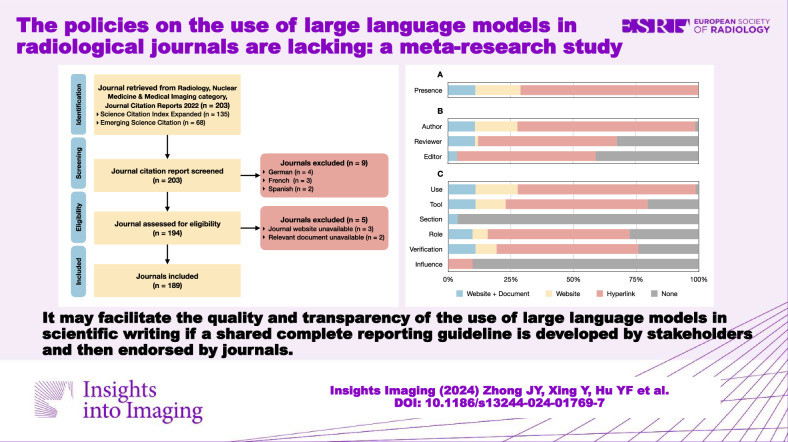

## Introduction

The generative large language model (LLM) is one of the emerging artificial intelligence techniques that typically employ deep neural networks to process a large scale of natural language data, and has presented potential in a broad spectrum of clinical tasks in the medical field [1], especially radiology [2–4]. The LLMs are employed to convert and explain the radiological reports [5, 6], to automatically extract and mine data from radiological reports [7, 8], and to optimize the clinical practice according to radiological reports [9, 10]. In addition to the remarkable potential of LLMs in the radiological field, the LLMs are used to generate scientific papers themselves [11]. The LLMs are considered as a helpful assistant in scientific writing with the ability to generate contents hard to indistinguishable from the writing of a medical researcher. However, it has limitations including potential bias, outdated data sources, insufficient transparency, and inclusion of inaccurate or inexistent information [12]. There is an increasing number of papers addressing the ethics of declaring the LLM use in medical academic writing [13–17], but the LLM use in scientific writing may not be always reported by the authors without clear policies or specific reporting guidelines.

**Fig. 1:**
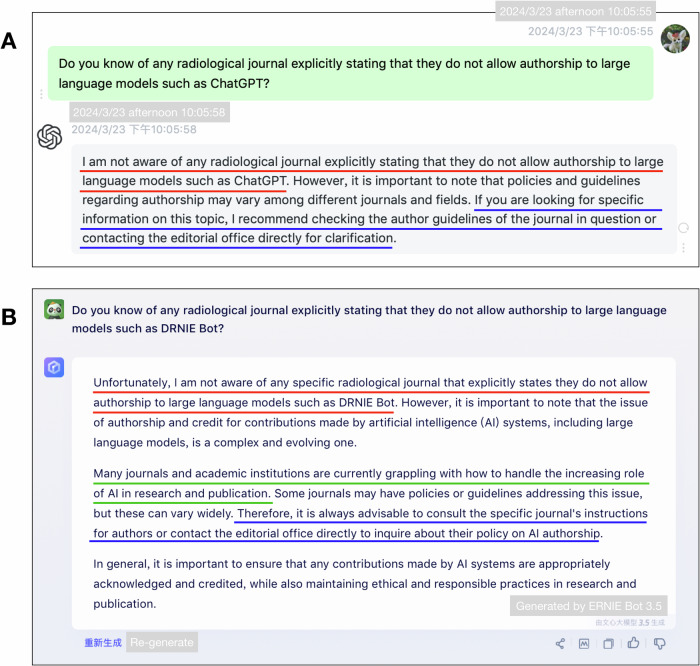
Conversations with LLMs on the topic of authorship policy of journals. These two screen captures showed conversations with (**A**) ChatGPT (ChatGPT-3.5-Turbo, OpenAI, https://openai.com/chatgpt) and (**B**) ERNIE Bot (ERNIE V2.5.4, Baidu, https://yiyan.baidu.com), respectively, performed at 22:00–22:10 (UTC + 8) on March 23, 2024. Both LLMs responded using plausible-sounding and grammatically correct sentences. They did not know the policy on the authorship of LLMs in any radiological journals (red line). However, they both suggest in the conversation that to check the policies of the specific journal (blue line). The ERNIE Bot even pointed out that journals and academic institutions are currently grappling with how to handle the increasing role of artificial intelligence in research and publication—just like our study (green line). The Chinese in the figure has been translated into English

There is an increasing number of papers discussing the potential and pitfalls of LLMs in scientific writing [18–25], but the reporting guideline of LLM use in medical research is still under development [26]. The reporting guidelines are documents that guide authors to transparently report a specific type of research [27]. Without complete and accurate reporting of the LLM use, the stakeholders may find it hard to differentiate the contents written by human authors from those generated by LLMs. As a result, it led to difficulties in the evaluation of the validity of a study, and the optimal application of the evidence [28, 29]. It is necessary to promote the use of the reporting guidelines to encourage complete reporting [30–34]. Nevertheless, the endorsement of the general reporting guidelines is still insufficient [35–37], and the implementation of the reporting guidelines for the application of artificial intelligence is even worse [38, 39]. If we ask the LLMs about the policy on their authorship in radiological journals, they will suggest we check the policies on LLM use in specific radiological journals by ourselves (Fig. 1). Here, we accepted their suggestions, and investigated the policies on the LLM use in radiological journals to provide insights for the establishment and promotion of a reporting standard for it. A shared reporting standard for the LLM use may allow a more reasonable, fair, and critical process for the authors, reviewers, and editors, to evaluate the papers whether they used LLMs or not.

As one of the medical fields that accepted and applied LLMs the earliest [5–10], we supposed that the radiological journals are much more likely to present their policies on LLM use. Therefore, the aim of our study was to evaluate whether and how the radiological journals present their policies on the LLM use, and identify the journal characteristic variables that are associated to the presence.

## Methods

### Study design

We performed a cross-sectional meta-research study [40–44]. We registered and uploaded relevant materials on Open Science Framework (https://osf.io/tpxkn/). The protocol for this study was drafted a priori and is available in Supplementary Note S1. Ethical approval or written informed consent was not required for this study because no human or animal subjects were included in this study. Since the reporting guideline for the meta-research study is under development [45], we reported our study in accordance with similar meta-research studies concerning journal policies [35–37]. Our review group consists of members with diverse backgrounds and knowledge from multiple disciplines to allow a balanced point of view for our study.

### Journal selection

We retrieved the journals in the Science Citation Index Expanded, and Emerging Science Citation Index, in Radiology, Nuclear Medicine and Medical Imaging Category, 2022 Journal Citation Reports via Clarivate on 20 December 2023 [46]. The journals were screened for eligibility by two independent reviewers, according to the exclusion criteria: (1) journals in non-English languages and (2) instructions for submission not available for assessment. Any discrepancies were resolved by discussion or consulting with the review group.

### Data extraction

We directly exported the following bibliometrics information of included journals via Clarivate [46]: journal name, journal abbreviation, 2022 journal impact factor (JIF), the JIF quartile, citable items, and total citations. The official website address of each journal was recorded, and the following items were extracted from the website of each journal: publication region, publication institution or publisher, publication frequency, type of access, whether the journal is only in the Radiology, Nuclear Medicine and Medical Imaging Category, and whether the journal is the official journal of an academic society. The data extraction was carried out by two independent reviewers from 22 December 2023 to 23 December 2023. Any discrepancies were resolved by discussion or consulting with the review group.

### Policy assessment

The assessment of policies on LLM use in radiological journals was performed according to a draft list of items and explanations for reporting standards for the application of LLMs [26], since there is no such guideline so far. We assessed (1) whether the journal presents its own policy on LLM use, (2) whether the journal presents the policy for the authors, the reviewers, and the editors, respectively, and (3) whether the journal presents the policy in terms of six potential reporting items: the usage of LLM, the name of LLM, the section that used LLM, the role of LLM, the verification of LLM, and the potential influence of LLM. The items, explanations, and examples of policy assessment are presented in Supplementary Note S2. We also reported the LLM use in the current study according to the six potential reporting items in Supplementary Note S3. The policy of each journal was assessed by two independent reviewers from 26 December 2023 to 31 December 2023. Any discrepancies were resolved by discussion or consulting with the review group.

### Statistical analysis

We performed the statistical analysis using R language version 4.1.3 within RStudio software version 1.4.1106. All the statistical tests were two-sided with an alpha level of 0.05, unless stated otherwise. We first descriptively summarized the data. The journals that presented their policies on LLM use were considered positive, while those that did not were treated as negative. We compared journal characteristics between the positive and negative groups. We evaluated the potential factors associated with the presence of policies on LLM use using univariate logistic regression with an alpha level of 0.10. The factors were included in the multivariate logistic regression if they were considered to be potentially associated with the presence of policies on LLM use. Multiple logistic regression analysis was used to estimate the adjusted odds ratio and 95% confidence interval. All the data generated and analyzed in this study is available in the Supplementary Data Sheet.

## Results

### Journal inclusion

**Fig. 2:**
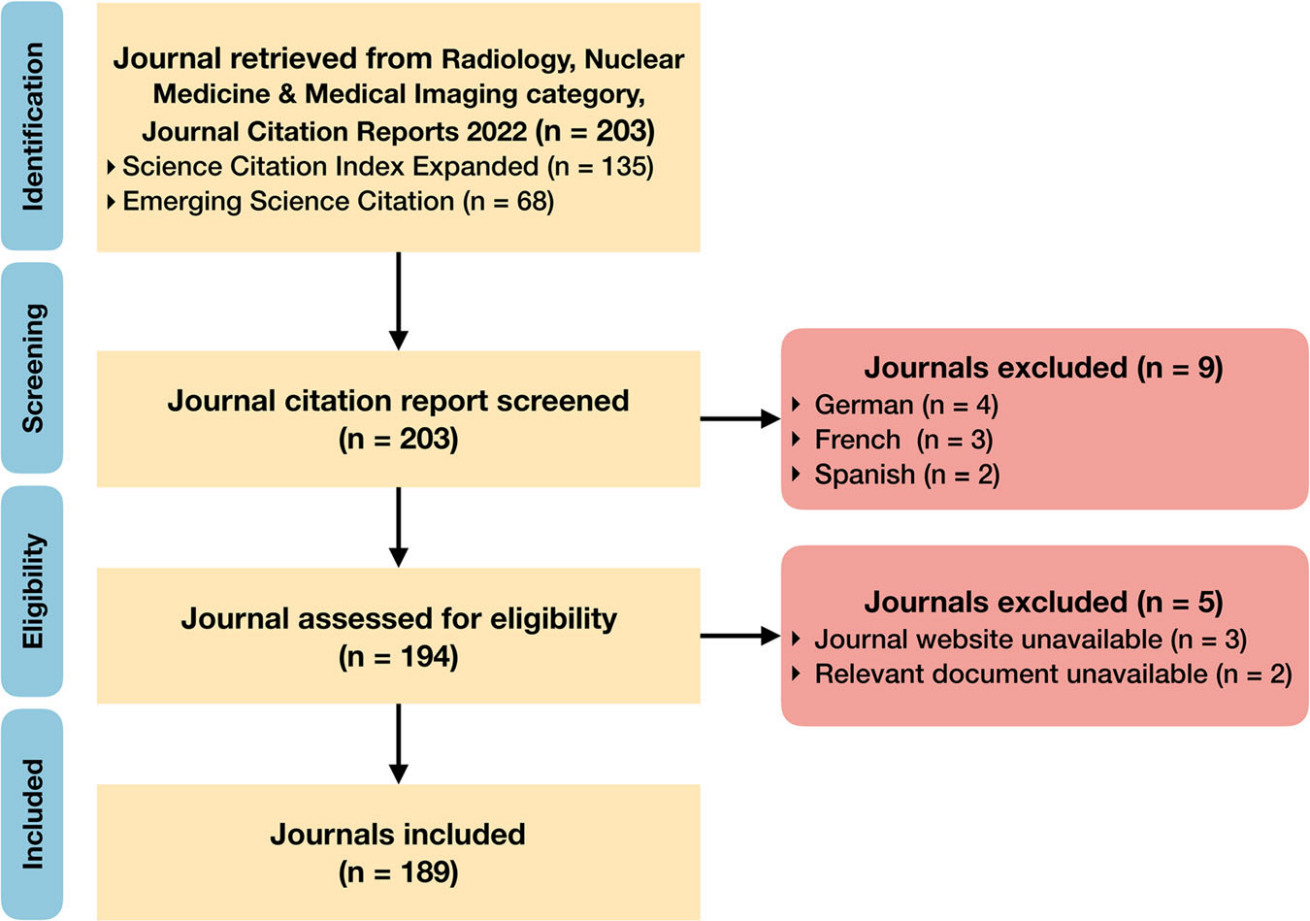
Flowchart of screening and inclusion of radiological journals

There were 135 and 68 journals in the lists of the Science Citation Index Expanded, and Emerging Science Citation Index, in Radiology, Nuclear Medicine and Medical Imaging Category, 2022 Journal Citation Reports, respectively. We excluded nine non-English journals, three journals without available websites for assessment, and two invited-only journals without publicly available instruction for submission. Finally, we included 189 radiological journals in total (Fig. 2).

### Journal characteristics

The mean ± standard deviation, median (range) of JIF was 3.0 ± 2.6, 2.4 (0.10–19.7) (Table 1). The mean ± standard deviation, median (range) of citable items and total citations were 131.1 ± 127.4, 87.0 (9.0–902.0) and 6136.3 ± 12,436.3, 1828.0 (13.0–129,835.0), respectively. The journals had more likely belonged to no JIF quartile (33.9%, 64/189), published by Springer (22.8%, 43/189), from North America (46.6%, 88/189), with a frequency of less than six issues per year (44.4%, 84/189) and a hybrid publishing model (61.9%, 117/189). Most of the journals were only in the Radiology, Nuclear Medicine, and Medical Imaging category (60.3%, 114/189), and were owned by an academic society (69.3%, 131/189).

**Table 1:** Characteristics of included radiological journals

Characteristics	All, (*N* = 189)	Present, (*N* = 83)	Not present, (*N* = 106)	*p* value
2022 JIF, mean ± SD, median (range)	3.0 ± 2.6, 2.4 (0.10–19.7)	3.6 ± 2.9, 3.1 (0.3–19.7)	2.5 ± 2.1, 2.1 (0.1–10.6)	0.002
Citable items, mean ± SD, median (range)	131.1 ± 127.4, 87.0 (9.0–902.0)	154.8 ± 156.3, 95.0 (16.0–902.0)	112.7 ± 96.0, 83.5 (9.0–450.0)	0.033
Total citations, mean ± SD, median (range)	6136.3 ± 12,436.3, 1828.0 (13.0–129,835.0)	8395.0 ± 16,707.2, 2624.0 (63.0–120,835.0)	4367.7 ± 7191.3, 1381.5 (13.0–3464.0)	0.043
JIF quartile, *n* (%)				0.162
n.a.	64 (33.9)	24 (28.9)	40 (37.7)	
Q1	33 (17.5)	20 (24.1)	13 (12.3)	
Q2	35 (18.5)	17 (20.5)	18 (17.0)	
Q3	32 (16.9)	14 (16.9)	18 (17.0)	
Q4	25 (13.2)	8 (9.6)	17 (16.0)	
Publisher, *n* (%)				< 0.001
Springer and BMC	43 (22.8)	15 (18.1)	28 (26.4)	
Elsevier	42 (22.2)	39 (47.0)	3 (2.8)	
Society	26 (13.8)	10 (12.0)	16 (15.1)	
Wiley and Hindawi	16 (8.5)	4 (4.8)	12 (11.3)	
Lippincott Williams & Wilkins	13 (6.9)	1 (1.2)	12 (11.3)	
Others	49 (25.9)	14 (16.9)	35 (33.0)	
Region, *n* (%)				0.234
North America	88 (46.6)	42 (50.6)	46 (43.4)	
Europe	74 (39.2)	34 (41.0)	40 (37.7)	
Asia	24 (12.7)	6 (7.2)	18 (17.0)	
Africa	3 (1.6)	1 (1.2)	2 (1.9)	
Publication frequency, *n* (%)				0.736
< 6 issue/year	84 (44.4)	39 (47.0)	45 (42.5)	
6–12 issue/year	53 (28.0)	21 (25.3)	32 (30.2)	
≥ 12 issue/year	52 (27.5)	23 (27.7)	29 (27.4)	
Publishing model, *n* (%)				0.852
Hybrid	117 (61.9)	52 (62.7)	65 (61.3)	
Open	72 (38.1)	31 (37.3)	41 (38.7)	
Only in radiology category, *n* (%)				0.359
Yes	114 (60.3)	47 (56.6)	67 (63.2)	
No	75 (39.7)	36 (43.4)	39 (36.8)	
Official journal, *n* (%)				0.640
Yes	131 (69.3)	59 (71.1)	72 (67.9)	
No	58 (30.7)	24 (28.9)	34 (32.1)	

*JIF* journal impact factor, *n.a.* not applicable, *Q1–Q4* the first to the fourth JIF quartile, *SD* standard deviation

### Policies on the LLM use

**Fig. 3:**
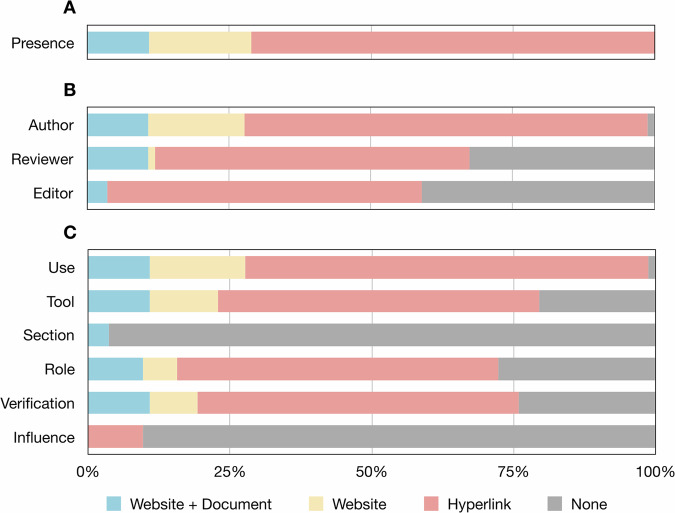
Bar plots of the location where the journals present their policies. There were 83 included journals that presented their policies on the use of LLMs. **A** The presence of the policies. **B** The presence of the policies for the author, the reviewer, and the editor, respectively. **C** The presence of the policies according to six potential reporting items

Less than half of the included radiological journals presented their policies on LLM use (43.9%, 83/189) (Table 2). The contribution of the publisher was different between the present and not present groups (*p* < 0.001) (Table 1). The policies were more likely to be presented for the authors (43.4%, 82/189), followed by those for the reviewers (29.6%, 56/189) and the editors (25.9%, 49/189). The aspects mentioned in the policies were whether the paper used LLMs (43.4%, 82/189), the name and other details of used LLMs (34.9%, 66/189), the verification of contents generated by LLMs (33.3%, 63/189), the role of LLMs in the writing process (31.7%, 60/189), the potential influence of LLMs on the paper (4.2%, 8/189), and the sections that used LLMs (1.6%, 3/189) in descending order. The journals preferred to present their policies on LLM use by providing a hyperlink to the common policy of the publisher (71.1%, 59/83) than by directly updating their own policies on the journal website (18.1%, 15/83) or by further publishing special documents on this issue (10.8%, 9/83) (Fig. 3). Representative examples for the presence of policies on the LLM use are available in Supplementary Note S4.

**Table 2:** Presence of policies on the use of LLMs in radiological journals

Presence of policies, *n* (%)	All, (*N* = 189)	Present, (*N* = 83)
Presence	83 (43.9)	83 (100.0)
Role		
Author	82 (43.4)	82 (98.8)
Reviewer	56 (29.6)	56 (67.5)
Editor	49 (25.9)	49 (59.0)
Six potential items		
Item 1: use	82 (43.4)	82 (98.8)
Item 2: tool	66 (34.9)	66 (79.5)
Item 3: section	3 (1.6)	3 (3.6)
Item 4: role	60 (31.7)	57 (72.3)
Item 5: verification	63 (33.3)	63 (76.0)
Item 6: influence	8 (4.2)	8 (9.6)

### Factors associated with the presence of policies on LLM use

Compared to journals published by Springer and BMC, the journals published by Elsevier were more likely to present their policies on LLM use (adjusted odds ratio 23.756, 95% confidential interval: 6.072–92.946, *p* < 0.001) (Table 3). The association between the presence of policies on LLM use and other factors was not found.

**Table 3:** Factors associated with the presence of policies on the use of LLMs in radiological journals

Variable grouping	Univariable logistic analysis			Multivariable logistic analysis		
	OR	95% CI	*p* value	OR	95% CI	*p* value
JIF quartile						
n.a.	1.000	1.000–1.000				
Q1	2.564	1.082–6.074	0.032	1.964	0.658–5.863	0.227
Q2	1.574	0.684–3.624	0.286	1.276	0.467–3.487	0.635
Q3	1.296	0.547–3.071	0.555	1.162	0.410–3.295	0.777
Q4	0.784	0.294–2.092	0.627	0.825	0.251–2.714	0.752
Publisher						
Springer and BMC	1.000	1.000–1.000				
Elsevier	24.267	6.410–91.870	< 0.001	23.756	6.072–92.946	< 0.001
Society	1.167	0.425–3.199	0.765	1.192	0.402–3.534	0.752
Wiley and Hindawi	0.622	0.171–2.269	0.472	0.628	0.159–2.475	0.506
Lippincott Williams & Wilkins	0.156	0.018–1.315	0.087	0.152	0.017–1.389	0.095
Other	0.747	0.309–1.803	0.561	0.884	0.351–2.221	0.792
Region						
North America	1.000	1.000–1.000				
Europe	0.931	0.501–1.730	0.821	0.977	0.429–2.224	0.955
Asia	0.365	0.132–1.007	0.052	0.827	0.271–2.527	0.739
Other	0.548	0.048–6.262	0.628	1.900	0.129–27.942	0.640
Publication frequency						
< 6 issue/year	1.000	1.000–1.000		n.a.		
6–12 issue/year	0.757	0.377–1.521	0.435	n.a.		
≥ 12 issue/year	0.915	0.457–1.834	0.803	n.a.		
Type of access						
Hybrid	1.000	1.000–1.000		n.a.		
Open	0.945	0.523–1.709	0.852	n. a.		
Only in radiology category						
Yes	1.000	1.000–1.000		n.a.		
No	1.316	0.732–2.366	0.359	n.a.		
Official journal						
Yes	1.000	1.000–1.000		n.a.		
No	0.861	0.461–1.610	0.640	n.a.		

*CI* confidence interval, *JIF* journal impact factor, *n.a.* not applicable, *OR* odds ratio, *Q1–Q4* the first to the fourth JIF quartile

## Discussion

Our study indicated that the policies on LLM use in radiological journals are lacking. We believe that such a policy is necessary, in order to enhance the transparency of the LLM use in the radiological academic community. In our study, less than half of the included radiological journals present their policies on LLM use. The policies were presented mostly for the authors, and followed by those for the reviewers and the editors. In the policies for authors, the aspects of the usage, the name, the verification, and the role of LLMs, were mentioned by about one-third of the journals, while the topics of the potential influence of LLMs, and the section that used LLMs, were seldomly touched. The publisher is associated with the presence of the policies on the LLM use.

An investigation of the top fifty radiological journals found that nearly half of these leading radiological journals did not provide any policy on LLM use [17]. Our study showed that only less than half of the radiological journals presented their policies on LLM use, indicating the gap in the recognition and regulation of LLM use in the radiological academic community. Most of the radiological journals with explicit policies referenced the common guidelines of major publishers [47–51], and only a few radiological journals presented their own policies in their instructions for submission or by editorials [52–55]. This is consistent with our findings that the publisher is associated with the presence of policies on LLM use. In those without their own policy, not all journals updated their websites with hyperlinks to the common guidelines on the LLM use of their publishers, resulting in a further reduction in the proportion of the presence of related policies. Further, journals having explicit policies provide hyperlinks to the publisher’s policies in varying places [17], which potentially obstructs authors from the relevant information.

The policies mainly discussed the issue of LLM use in scientific writing for the authors [47–55]. The major publishers and journals agreed that the LLMs should not be listed as authors for a paper since they could not take responsibility or have accountability for papers. Among these policies, some strictly limited the use of the LLMs in scientific writing for the improvement of the language and readability of the paper [48, 52], while the others only asked for appropriate disclosure for the LLM use. Notably, with the rapidly evolving ability of the LLMs, the images and videos from the generative artificial intelligence tools have been discussed in these policies [47, 48, 50, 52]. Although these images and videos are currently not allowed to be published due to legal copyright and research integrity issues, this reminds us to expand the scope of policies and reporting guidelines beyond texts in scientific writing to multimodal forms of information expression. In addition to the policies from the publishers, the radiological journals presented their policies in various documents [52–55], and offered various locations for the declaration of the LLM use [17]. Although efforts have been taken to reach a shared point of view on this issue, it seems that a standardized approach for addressing LLM use has not been established by the journals yet [17].

A reporting guideline for LLM use is under development, in order to enhance the transparency of LLM use in medical research [26]. We assessed the policies according to this paper in six potential reporting items. However, none of these policies fulfilled these six items to allow a relatively complete report of the LLM use. The potential influence of LLMs [49], and the exact section that used LLMs [55], were most less discussed. It is not only critical to establish policies on the LLM use in journals, but also important to develop and endorse a complete guideline for authors to cover the necessary items. The complete and appropriate report of the LLM use may allow the reviewers and editors to perform a fairer peer review process and make reasonable decisions on the paper. Furthermore, the papers written with LLMs may suffer the outdated or inaccurate data, inappropriate prompts, and unstable responses [56]. The stakeholders can benefit by the optimal reporting of the LLM use, to make better validity evaluations on the evidence. The quality of studies using LLMs is potentially influenced by whether the generated content has been well-confirmed and critically revised. It is difficult to forbid the LLMs in scientific writing. It may be wiser to encourage the authors, the reviewers, and the editors to use them smartly, with mandatory reporting. In addition to the potential influence on the quality of the study, the underlying issue of the use of LLM in scientific writing is the ethical problem. The lack of policies may affect the fact that the line between what should and should not be done is still blurred. Therefore, we highlighted the need to create regulations that control these procedures. Jeblick et al [6] recently published a paper on the ability of ChatGPT to simplify radiological reports, whose title was generated by ChatGPT. This title made the paper more interesting while not compromising its scientific robustness. Alike scientific writing, LLM use can be very beneficial in radiological report writing. It is urgent to revisit our position in writing and signing the reports with the rapid involvement of these techniques. The LLM use in report writing may provide insights for guideline development for the LLM use in scientific writing. On the other hand, Hamm [23] wrote an editorial to introduce the European Society of Radiology journals editors’ joint statement on guidelines for LLM use, and emphasized at the end that the editorial was not written with the help of LLMs but with input from the editorial staff. This extra note once again reminded us that it is human insights always the most essential element in scientific writing.

Besides the policies on the use of the LLM for authors, we further evaluated and found an even lower percentage of journals presenting their policies on the LLM use for the reviewers and the editors. Less than one-third of the journals declared their policies for reviewers. The journals believe that critical thinking and original assessment are the keys to peer review, which is still lacking in the LLMs [48, 52–55]. Further, there is a concern technology that it may generate conclusions on the paper with an incorrect, incomplete, or biased point of view. Another reason for regulating the use of LLMs in peer review is that their use may violate the confidentiality and proprietary rights of the author, as well as data privacy rights if the paper contains personally identifiable information. The reviewers are valued for their role as human oversight for the review process, and are responsible and accountable for the review report [49, 52, 55]. However, the reviewers are allowed to use tools that do not violate the confidentiality policy with appropriate reporting [52, 55]. About one-fourth of the policies were written for the editors, and asked them to fulfill the confidentiality obligations, and to report potential violations against the policies [48, 49, 55]. It is still unclear how these policies for reviewers and editors may influence the peer review process and editorial decision-making on the papers.

Our study has the following limitations. First, our study only included radiological journals. Indeed, the editors of radiology journals have discussed and reached a consensus on the influence of artificial intelligence-assisted technology on biomedical publishing [52, 55]. Nevertheless, it is necessary to evaluate the policies on the LLM use in medical journals. Second, our study was a cross-sectional study that relied on websites and online documents. As a rapidly developing field, the journals and publishers may adapt their policies if necessary. Additional instructions may appear during the paper submission for authors, the review process for reviewers, and the editorial systems for editors. An updated study with more comprehensive documents should be conducted in the future. Finally, we only assessed whether the journals presented their policies on LLM use. Since there is currently no guideline for reporting LLM use, we could not rate the level of endorsement of such a guideline [35, 36, 38], but evaluate the aspects mentioned in the policies. Nonetheless, our study showed the *status quo* of journal policies on LLM use, which may help the development of a reporting standard for the application of LLMs in medical research [26].

In summary, our study showed that the percentage of radiological journals that present their own policies on LLM use is low. A reporting guideline is necessary to promote the reporting transparency of the LLM use in medical research.

### Supplementary information

ELECTRONIC SUPPLEMENTARY MATERIAL

Supplementary Data Sheet

## Data Availability

Raw data collected within the study are published on Open Science Framework (https://osf.io/tpxkn/).

## Abbreviations

JIF: Journal impact factor
LLM: Large language model

## Declarations

### Authors' information

The abstract of this article entitled “The policies on the use of large language models in radiological journals are lacking: a meta-research study” (C-22718) has been accepted as a digital poster, EPOS Radiologist (scientific), on European Congress of Radiology 2024 (10.26044/ecr2024/C-22718). The presenting author of this abstract is Dr. Jingyu Zhong.
