# Supplementary material for: The policies on the use of large language models in radiological journals are lacking: a meta-research study
Source: Insights Imaging. 2024 Aug 1;15:186. doi: 10.1186/s13244-024-01769-7 (PMC11294318; doi:10.1186/s13244-024-01769-7)
Supplement: Supplementary file 1 — ELECTRONIC SUPPLEMENTARY MATERIAL [file 13244_2024_1769_MOESM1_ESM.pdf]

The policies on the use of large language models in radiological journals are lacking: a meta-research study
ELECTRONIC SUPPLEMENTARY MATERIAL

List of Supplementary Material

Supplementary Note S1 Study protocol
Supplementary Note S2 Assessment criteria for policies on the use of large language models with examples in radiological journals
Supplementary Note S3 Reporting of the use of large language models in current study
Supplementary Note S4 Representative examples for the presence of policies on the use of large language models in radiological journals

Supplementary Note S1 Study protocol

Study rationale

The emerging large language models (LLM) are artificial intelligence systems that learn from vast training data up to billions of words to understand how words are used with each other, and then apply these recognized patterns to complete natural language processing tasks [1]. This language model can respond to queries and compose diverse written content, spanning from articles, social media posts, and essays to code and emails. Researchers have employed large language models to draft scientific papers, especially in the field of radiology [2-6], which is one of the most used field among medical specialties [7]. The use of such tools has increased the researchers' efficiency and productivity to some extent. However, there may also be some issues and limitations associated with this approach [8]. First, the data used to train large language models like ChatGPT may have a time lag, meaning the generated content may not be up-to-date or accurate. Second, the quality of the questions posed to large language models greatly affect the quality of its responses, and even slightly different prompts can yield inconsistent results. Because of these challenges, authors should not rely on ChatGPT or similar tools alone and always confirm and critically revise the generated content.

The radiological journals have presented their policies on the use of large language models. On the websites of Radiology (https://pubs.rsna.org/page/policies#llm), and European Radiology (https://www.european-radiology.org/publication-ethics/#guidelines), there are "Guidelines for the use of large language models". The *Korean Journal of Radiology* has published "Authorship policy of the *Korean Journal of Radiology* regarding artificial intelligence large language models such as ChatGTP" [9], and "Use of generative artificial intelligence, including large language models such as ChatGPT, in scientific publications: policies of *KJR* and prominent authorities" [10]. However, it is unclear whether and how are the policies on the use of large language models in radiological journals. Therefore, we designed this study to assess the policies on the use of large language models by authors, reviewers, and editors in radiological journals, and explore associated journal characteristic variables.

Study design

Our study is a cross-sectional meta-research study [11-15]. We did not register the study protocol since there were no appropriate platform. However, we drafted this protocol for this study *a priori*. The ethical approval or written informed consent was not required for this study because no human or animal subjects have been included in this study.

Study group

Our study group is consisted of members with diverse background and knowledge from multiple disciplines all with have experience in manuscript drafting and publishing: (1) five radiologists with 3 to 30 years of experience in radiology diagnosis and research, in which three of them have 3 to 6 months experience in prompt fine-turning of large language models, one serves as a reviewer for an evidence-based medicine journal (*Chinese Journal of Evidence-Based Medicine* [Journal in Chinese], http://www.cjebm.com/journal/zgxzyxzz), as well as serve as an editorial board member for radiological journals (*European Radiology,* https://www.springer.com/journal/330/; *BMC Medical Imaging*, https://bmcmedimaging.biomedcentral.com); (2) a methodologist in epidemiology and population health who provides methodological and statistical suggestions for this study; (3) a biomedical engineer with experience in radiology-related research; (3) an orthopedist with 5 years of experience in clinical practice and research whose interest is sports medicine; (4) a medical oncologist with 5 years of experience in clinical practice and research whose interest is lung cancer; (5) a surgeon with 5 years of experience in clinical practice and research whose interest is pancreatic disease; (6) a pathologist with 2 years of experience in clinical practice and research whose current interest is bone tumor, and has formerly worked as a gynecology and obstetrics doctor for 3 years; (7) a dermatologist with 5 years of experience in clinical practice and research whose interest is cutaneous hemangioma; (8) an expert in pharmacovigilance with 2 years of experience, and has formerly worked as an ophthalmologist for 3 years; (9) a full-time editor of a medical journal (*Journal of Diagnostics and Concepts and Practice* [Journal in Chinese], https://www.qk.sjtu.edu.cn/jdcp/CN/1671-2870/home.shtml) with 15 years of experience; (10) a MR collaboration scientist doing technical support with 10 years of experience, and has 1 year of experience in prompt fine-turning of large language models.

The diversity of our study group allows a balanced point of view for our study, and better dissemination of our study via conference abstracts, journal articles, and oral presentations to the stakeholders.

Study purpose

This study aims to assess the policies on the use of large language models by authors, reviewers, and editors in radiological journals, and explore associated journal characteristic variables.

Journal selection
The journals from the Science Citation Index Expanded (SCIE) and Emerging Science Citation Index (ESCI) lists of Radiology, Nuclear Medicine & Medical Imaging category, of the 2022 Journal Citation Reports were identified via Clarivate (https://jcr.clarivate.com/jcr/home) by a member with experience in literature searching. The journals will be screened and assessed for eligibility by two independent members. Any discrepancies will be resolved by discussion or consulting with the review group.

The journals will be screened for eligibility according to the exclusion criteria:
(1) journals in non-English languages. The publication language of journal will be defined according to the Journal Citation Reports. If the publication language of journal is multi-language, we will further confirm the publication language of this journal to decide whether the journal should be included or excluded. If the publication language of journal is non-English (e. g., German, French), the journal will be excluded. If the publication language of journal is English, the journal will be included.
(2) journals website not available for assessment. The journal will be excluded if the official website is unavailable, or the instruction for authors, author guidelines, peer reviewer guidance, editorial policies, or other relevant directions for authors of a journal is unavailable.

Data extraction
The bibliometrics information of included journals will be downloaded directly from the from the Science Citation Index Expanded (SCIE) and Emerging Science Citation Index (ESCI) lists of Radiology, Nuclear Medicine & Medical Imaging category, of the 2022 Journal Citation Reports were identified via Clarivate (https://jcr.clarivate.com/jcr/home) by a member with experience in literature searching. The homepages of included journals will be identified by the same member. The data extraction will be conducted and cross-checked by two independent members. Any discrepancies will be resolved by discussion or consulting with the review group.

The following data will be extracted:
(1) Bibliometrics information: journal name, JCR abbreviation, ISSN, eISSN, 2022 journal impact factor (JIF), the JIF quartile (Q1, Q2, Q3, Q4, n. a. for SCIE journals), JIF rank, total citations, citable items, etc.
(2) Journal characteristics: publication region (North America, European, Asia), publication institution/publisher (Elsevier, Lippincott Williams & Wilkins, Springer, Wiley, Oxford, etc.), publication language (only English available), publication frequency (monthly, bi-monthly, quarterly, etc.), type of access (conventional, open, or hybrid), whether the journal is only in the Radiology, Nuclear Medicine & Medical Imaging category (yes, no), whether the journal is owned by a professional society or institute (yes, no), and official homepage website address of each journal.

Policy assessment
When utilizing ChatGPT or similar chatbots in writing papers or conducting medical research, it is important to adhere to reporting standards and provide detailed information about the process. Although the World Health Organization Collaborating Centre for Guideline Implementation and Knowledge Translation proposes to develop reporting guidelines for the use of ChatGPT and similar conversational tools in medical research writing, there is currently no reporting guideline for the use of large language models. Therefore, we decided to apply the six potential reporting items proposed by Luo et al [8]. The policies on the use of large language models of included journals will be conducted by two independent members. Any discrepancies will be resolved by discussion or consulting with the review group.

The policies on the use of large language models will be assessed.
(1) The journal will be considered "yes" for presenting the policy on the use of large language models, if there is a "Guidelines for the use of large language models by authors, reviewers, and editors" (extracted from website of *European Radiology*, https://www.european-radiology.org/publication-ethics/#guidelines), or similar declarations or documents.
(2) The journal will be considered "yes" for presenting the policy on the use of large language models by (2.1) authors, (2.2) reviewers, and (2.3) editors, respectively, if there is "Authors are fully responsible for correctly labelling and disclosing which parts of their work has been created by or in assistance of AI" for (2.1) author (extracted from website of *European Radiology*, https://www.european-radiology.org/publication-ethics/#guidelines), or "Reviewers are forbidden from using large language models for the primary purpose of generating review comments" for (2.2) reviewers (extracted from Editorial of *Korean Journal of Radiology*, Park SH. Use of generative artificial intelligence, including large language models such as ChatGPT, in scientific publications: policies of KJR and prominent authorities. Korean J Radiol 2023;24(8):715-718. https://doi.org/10.3348/kjr.2023.0643), or "Reviewers and editors are obliged to confidentiality and should not upload manuscripts to software or AI-assisted tools where confidentiality cannot be assured" for both (2.2) reviewers and (2.3) editors (extracted from website of *European Radiology*, https://www.european-radiology.org/publication-ethics/#guidelines), or similar declarations or documents.
(3) The following six potential reporting items proposed by Luo et al [7] will be assessed: use, tool, section, role, verification, and influence. (3.1) Did you use ChatGPT or similar software/tools to assist in writing the manuscript? (3.2) Please specify the AI tool and its version that was used in writing the manuscript. (3.3) Please report the specific section of the manuscript where ChatGPT or similar tools were used. (3.4) Please describe the role of ChatGPT in assisting the manuscript writing process. (3.5) Please report whether the content generated by ChatGPT was verified and/or modified. If not, please explain the reason. (3.6) Please report whether and how the ChatGPT-generated content can be expected to have influenced the overall conclusions and accuracy of the research.

Statistical analysis
The statistical analysis will be performed with R language version 4.1.3 (https://www.r-project.org/) within RStudio software version 1.4.1106 (https://www.rstudio.com/) using relevant packages by a member under supervision of a statistical expert. All of the statistical tests were 2-sided. The alpha level for statistically significance is set at 0.05, if not stated otherwise.

The continuous variables will present as mean ± standard deviation (SD) if they showing a normal distribution, or median (interquartile range, IQR) if they showing a non-normal distribution. The categorical variables will present as frequency distribution (n) and percentages (%). To assess intergroup differences, the independent sample t test was used for continuous variables showing a normal distribution, the Mann–Whitney U test for non-normally distributed variables. The chi-square test for categorical variables, and Fisher's exact test for those with small sample sizes. To assess multigroup differences the analysis of variables will be used for those without heterogeneity of variance, and the Kruskal–Wallis H test will be used for those with heterogeneity of variance.

The univariate and multivariate logistic regression will be only performed for whether the journal presents policy on the use of large language models. The factors will be included into the multivariate logistic regression if the factor is considered to be potentially associated with whether the journal presents policy on the use of large language models in the univariate logistic regression. The alpha level for univariate logistic regression will set at 0.10. Multiple logistic regression analysis will be used to estimate adjusted ORs and 95% CIs of whether factors are associated with the reporting guidelines endorsement. The alpha level for multivariate logistic regression will set at 0.05.

Odds ratios (ORs) and 95% confidence intervals (CIs) will be calculated to investigate whether (1) JIF quartile (Q1, Q2, Q3, Q4, and n. a. for SCIE journals); (2) publication institution/publisher (Elsevier, Lippincott Williams & Wilkins, Springer, Wiley, Oxford, etc.); (3) region (North America, Europe, Asia); (4) publication frequency (monthly, bi-monthly, quarterly, etc.); (5) type of access (conventional, open, or hybrid); (6) Only in Radiology, Nuclear Medicine & Medical Imaging category (yes, no); (7) official journal owned by societies (yes, no), are associated with whether the journal presents policy on the use of large language models.

Reporting and dissemination

We plan to report this study via peer-reviewed journals. One member will draft the original version of the manuscript. All the members will read and edit the manuscript critically. We plan to disseminate our study via conference abstracts, journal articles, and oral presentations.

Reference

1. Thirunavukarasu AJ, Ting DSJ, Elangovan K, Gutierrez L, Tan TF, Ting DSW. Large language models in medicine. Nat Med 2023;29(8):1930-1940. https://doi.org/10.1038/s41591-023-02448-8
2. Adams LC, Truhn D, Busch F, et al. Leveraging GPT-4 for post hoc transformation of free-text radiology reports into structured reporting: a multilingual feasibility study. Radiology 2023;307(4):e230725. https://doi.org/10.1148/radiol.230725
3. Jeblick K, Schachtner B, Dexl J, et al. ChatGPT makes medicine easy to swallow: an exploratory case study on simplified radiology reports. Eur Radiol. 2023. https://doi.org/10.1007/s00330-023-10213-1
4. Fink MA, Bischoff A, Fink CA, et al. Potential of ChatGPT and GPT-4 for data mining of free-text CT reports on lung cancer. Radiology 2023;308(3):e231362. https://doi.org/10.1148/radiol.231362
5. Ueda D, Mitsuyama Y, Takita H, et al. ChatGPT's diagnostic performance from patient history and imaging findings on the Diagnosis Please Quizzes. Radiology 2023;308(1):e231040. https://doi.org/10.1148/radiol.231040
6. Nowak S, Schneider H, Layer YC, et al. Development of image-based decision support systems utilizing information extracted from radiological free-text report databases with text-based transformers. Eur Radiol. 2023. https://doi.org/10.1007/s00330-023-10373-0
7. Barrington NM, Gupta N, Musmar B, et al. A Bibliometric analysis of the rise of ChatGPT in medical research. Med Sci (Basel) 2023;11(3):61. https://doi.org/10.3390/medsci11030061
8. Luo X, Estill J, Chen Y. The use of ChatGPT in medical research: do we need a reporting guideline? Int J Surg. 2023. https://doi.org/10.1097/JS9.0000000000000737
9. Park SH. Authorship policy of the *Korean Journal of Radiology* regarding artificial intelligence large language models such as ChatGTP. Korean J Radiol 2023;24(3):171-172. https://doi.org/10.3348/kjr.2023.0112
10. Park SH. Use of generative artificial intelligence, including large language models such as ChatGPT, in scientific publications: policies of KJR and prominent authorities. Korean J Radiol 2023;24(8):715-718. https://doi.org/10.3348/kjr.2023.0643
11. Ioannidis JP, Fanelli D, Dunne DD, Goodman SN. Meta-research: evaluation and improvement of research methods and practices. PLoS Biol. 2015;13(10):e1002264. https://doi.org/10.1371/journal.pbio.1002264
12. Puljak L. Methodological studies evaluating evidence are not systematic reviews. J Clin Epidemiol. 2019;110:98-99. https://doi.org/10.1016/j.jclinepi.2019.02.002
13. Puljak L. Methodological research: open questions, the need for 'research on research' and its implications for evidence-based health care and reducing research waste. Int J Evid Based Healthc. 2019;17(3):145-146. https://doi.org/10.1097/XEB.0000000000000201
14. Puljak L, Makaric ZL, Buljan I, Pieper D. What is a meta-epidemiological study? Analysis of published literature indicated heterogeneous study designs and definitions. J Comp Eff Res. 2020;9(7):497-508. https://doi.org/10.2217/cer-2019-0201
15. Mbuagbaw L, Lawson DO, Puljak L, Allison DB, Thabane L. A tutorial on methodological studies: the what, when, how and why. BMC Med Res Methodol. 2020;20(1):226. https://doi.org/10.1186/s12874-020-01107-7

Supplementary Note S2 Assessment criteria for policies on the use of large language models with examples in radiological journals

1. Present of policy
Description: Is there a policy on the use of LARGE LANGUAGE MODEL?
Explanation: If there is policy, guideline, declaration, statements, or similar documents present on the website of the journal or published on the journal, this item will be rated as "yes" regardless of the policy for authors, reviewers, or editors, i. e., this item will be rated as "yes" even there is only a policy for the editors but not for the authors.
Example 1:
"Guidelines for Use of Large Language Models"
(extracted from website of *Radiology*; https://pubs.rsna.org/page/policies#llm).
Example 2:
"Guidelines for the use of large language models by authors, reviewers, and editors"
(extracted from website of *European Radiology*; https://www.european-radiology.org/publication-ethics/#guidelines).
Example 3:
"Peer reviewers are required to maintain confidentiality regarding the manuscripts they review and must not divulge any information about a specific manuscript or its content to any third party without prior permission from the journal's editors, which may prohibit the uploading of the manuscript to software or other AI technologies where confidentiality cannot be assured. Reviewers should disclose to editors if and how AI technology is being used to facilitate their review and be aware that AI can generate authoritative-sounding output that can be incorrect, incomplete, or biased."
(extracted from website of *Quantitative Imaging in Medicine and Surgery,* https://qims.amegroups.org/pages/view/guidelines-for-reviewers).

2. Policies for authors, reviewers, and editors
2.1 Authors
Description: Is there a policy on the use of LARGE LANGUAGE MODEL by authors?
Explanation: If there is policy, guideline, declaration, statements, or similar documents present on the website of the journal or published on the journal for the authors, this item will be rated as "yes".
Example 1:
"AI or AI-assisted technologies do not qualify as authors and must not be listed as authors or co-authors. At submission, authors must disclose whether they used AI or AI-assisted technologies in the preparation of the manuscript. Authors may use LARGE LANGUAGE MODELs to assist with medical writing and for content editing to effectively communicate their work. All authors are responsible for any submitted material that includes AI-assisted technologies. AI-assisted technologies cannot distinguish between true and false information. Authors should be able to assert that there is no plagiarism in their paper, including in text and images produced by AI. The submission and publication of content/images created by AI, language models, machine learning, or similar technologies is discouraged, unless part of formal research design or methods, and is not permitted without clear description of the content that was created and the name of the model or tool, version and extension numbers, and manufacturer."
(extracted from website of *Radiology*; https://pubs.rsna.org/page/policies#llm).
Example 2:
"At submission, authors must disclose whether they used AI or AI-assisted technologies in the preparation of the manuscript. Authors who use such technology must clearly describe, in both the cover letter and the manuscript, how AI or AI-assisted technologies (LLMs, chatbots such as ChatGPT or Bard, and image creators) were used in the study and/or manuscript preparation. Authors should be transparent when AI-assisted technologies are used and provide information about their use. Authors may provide this information in the Materials and Methods section, the Acknowledgments section, or a relevant section of the manuscript (eg, figure legends for AI-generated figures). Authors should include specific details, such as the name and version of the AI tool, date of access, and name of the manufacturer or creator.
(extracted from Editorial of *Radiology*, Moy L. Guidelines for use of large language models by authors, reviewers, and editors: considerations for imaging journals. Radiology. 2023;309(1):e239024. https://doi.org/10.1148/radiol.239024).
Example 3:
"AI or AI-assisted tools do not qualify as authors, only humans do. Authors are fully responsible for the entire content of their work. Authors are fully responsible for correctly labelling and disclosing which parts of their work has been created by or in assistance of AI. AI-tools used to generate results must be described in detail in the methods section. AI-tools used for writing and content editing must be disclosed in the acknowledgements."
(extracted from website of *European Radiology*; https://www.european-radiology.org/publication-ethics/#guidelines).
Example 4:
"Authors must disclose at submission whether they used AI or AI-assisted technologies in their work."
(extracted from Editorial of ESR Journals, Hamm B, Marti-Bonmati L, Sardanelli F. ESR Journals editors' joint statement on Guidelines for the Use of Large Language Models by Authors, Reviewers, and Editors. Eur Radiol 2024. https://doi/org/10.1007/s00330-023-10511-8).

2.2 Reviewers
Description: Is there a policy on the use of LARGE LANGUAGE MODEL by reviewers?
Explanation: If there is policy, guideline, declaration, statements, or similar documents present on the website of the journal or published on the journal for the reviewers, this item will be rated as "yes".
Example 1:
"The ICMJE and WAME guidelines states that reviewers should not upload the manuscript to software or other AI technologies where confidentiality cannot be assured. Reviewers are trusted and required to maintain confidentiality throughout the manuscript review process."
(extracted from website of *Radiology*; https://pubs.rsna.org/page/policies#llm).
Example 2:
"Several journals have followed the ICJME and WAME guidelines and state that entering any part of the manuscript or abstract or the text of a review into a chatbot, language model, or similar tool is a violation of the journals' confidentiality agreement. The review process is valued for its human expert perspective and human oversight with decision-making in scholarly publication, including the need for accountability and human oversight. If a reviewer used an AI tool as a resource for their review in a way that does not violate the journal's confidentiality policy, then they must provide the name of the tool and how it was used."
(extracted from Editorial of *Radiology*, Moy L. Guidelines for use of large language models by authors, reviewers, and editors: considerations for imaging journals. Radiology. 2023;309(1):e239024. https://doi.org/10.1148/radiol.239024).
Example 3:
"Reviewers and editors are obliged to confidentiality and should not upload manuscripts to software or AI-assisted tools where confidentiality cannot be assured."
(extracted from website of *European Radiology*; https://www.european-radiology.org/publication-ethics/#guidelines).
Example 4:
"Reviewers and editors are obligated to confidentiality and should not upload manuscripts to software or other AI-assisted tools where confidentiality cannot be assured. Reviewers and editors are trusted and required to maintain confidentiality throughout the manuscript review process. Authors trust the reviewers and editors to protect their proprietary, sensitive, and confidential ideas. The use of AI-assisted tools may violate peer review confidentiality expectations, and several journals have followed the ICJME and WAME guidelines and state that entering any part of the manuscript or abstract or the text of your review into a chatbot, language model, or similar tool is a violation of the journals' confidentiality agreement. The review process is valued for its human expert perspective and human oversight with decision-making in scholarly publication, including the need for accountability and human oversight. If a reviewer or editor used an AI tool as a resource for his/her review in a way that does not violate the journal's confidentiality policy, he/she must provide the name of the tool and how it was used."
(extracted from Editorial of ESR Journals, Hamm B, Marti-Bonmati L, Sardanelli F. ESR Journals editors' joint statement on Guidelines for the Use of Large Language Models by Authors, Reviewers, and Editors. Eur Radiol 2024. https://doi/org/10.1007/s00330-023-10511-8).
Example 5:
"Reviewers are forbidden from using LARGE LANGUAGE MODELs for the primary purpose of generating review comments. The review process is valued for its human expert perspective, and substitution of this perspective with AI-generated inputs is not permitted. However, reviewers may use LARGE LANGUAGE MODELs or other AI tools to enhance the linguistic quality of their review comments (improve grammatical accuracy, rectify typographical errors, enhance formatting, ensure clarity, avoid demeaning or condescending tones, etc.)"
(extracted from Editorial of *Korean Journal of Radiology*, Park SH. Use of generative artificial intelligence, including large language models such as ChatGPT, in scientific publications: policies of KJR and prominent authorities. Korean J Radiol 2023;24(8):715-718. https://doi.org/10.3348/kjr.2023.0643).
Example 6:
"Peer reviewers are required to maintain confidentiality regarding the manuscripts they review and must not divulge any information about a specific manuscript or its content to any third party without prior permission from the journal's editors, which may prohibit the uploading of the manuscript to software or other AI technologies where confidentiality cannot be assured. Reviewers should disclose to editors if and how AI technology is being used to facilitate their review and be aware that AI can generate authoritative-sounding output that can be incorrect, incomplete, or biased."
(extracted from website of *Quantitative Imaging in Medicine and Surgery,* https://qims.amegroups.org/pages/view/guidelines-for-reviewers).
Example 7:
"Editors and Reviewers must uphold the confidentiality of the peer review process. Editors must not share information about submitted manuscripts or peer review reports with generative AI or LLMs such a ChatGPT. Reviewers must not use artificial intelligence tools to generate review reports, including but not limited to ChatGPT."
(extracted from policy of SAGE, https://us.sagepub.com/en-us/nam/chatgpt-and-generative-ai).

2.3 Editors
Description: Is there a policy on the use of LARGE LANGUAGE MODEL by editors?
Explanation: If there is policy, guideline, declaration, statements, or similar documents present on the website of the journal or published on the journal for the editors, this item will be rated as "yes".
Example 1:
"Reviewers and editors are obliged to confidentiality and should not upload manuscripts to software or AI-assisted tools where confidentiality cannot be assured."
(extracted from website of *European Radiology*; https://www.european-radiology.org/publication-ethics/#guidelines).
Example 2:
"Reviewers and editors are obligated to confidentiality and should not upload manuscripts to software or other AI-assisted tools where confidentiality cannot be assured. Reviewers and editors are trusted and required to maintain confidentiality throughout the manuscript review process. Authors trust the reviewers and editors to protect their proprietary, sensitive, and confidential ideas. The use of AI-assisted tools may violate peer review confidentiality expectations, and several journals have followed the ICJME and WAME guidelines and state that entering any part of the manuscript or abstract or the text of your review into a chatbot, language model, or similar tool is a violation of the journals' confidentiality agreement. The review process is valued for its human expert perspective and human oversight with decision-making in scholarly publication, including the need for accountability and human oversight. If a reviewer or editor used an AI tool as a resource for his/her review in a way that does not violate the journal's confidentiality policy, he/she must provide the name of the tool and how it was used."
(extracted from Editorial of ESR Journals, Hamm B, Marti-Bonmati L, Sardanelli F. ESR Journals editors' joint statement on Guidelines for the Use of Large Language Models by Authors, Reviewers, and Editors. Eur Radiol 2024. https://doi/org/10.1007/s00330-023-10511-8).
Example 3:
"Editors and Reviewers must uphold the confidentiality of the peer review process. Editors must not share information about submitted manuscripts or peer review reports with generative AI or LLMs such a ChatGPT. Reviewers must not use artificial intelligence tools to generate review reports, including but not limited to ChatGPT."
(extracted from policy of SAGE, https://us.sagepub.com/en-us/nam/chatgpt-and-generative-ai).
Example 4:
"A submitted manuscript must be treated as a confidential document. Editors should not upload a submitted manuscript or any part of it into a generative AI tool as this may violate the authors' confidentiality and proprietary rights and, where the paper contains personally identifiable information, may breach data privacy rights.
This confidentiality requirement extends to all communication about the manuscript including any notification or decision letters as they may contain confidential information about the manuscript and/or the authors. For this reason, editors should not upload their letters into an AI tool, even if it is just for the purpose of improving language and readability.
Peer review is at the heart of the scientific ecosystem and Elsevier abides by the highest standards of integrity in this process. Managing the editorial evaluation of a scientific manuscript implies responsibilities that can only be attributed to humans. Generative AI or AI-assisted technologies should not be used by editors to assist in the evaluation or decision-making process of a manuscript as the critical thinking and original assessment needed for this work is outside of the scope of this technology and there is a risk that the technology will generate incorrect, incomplete or biased conclusions about the manuscript. The editor is responsible and accountable for the editorial process, the final decision and the communication thereof to the authors."
(extracted from policy of Elsevier, https://www.elsevier.com/about/policies-and-standards/publishing-ethics#Authors, https://us.sagepub.com/en-us/nam/chatgpt-and-generative-ai).

3. Six potential reporting items for authors
Six potential reporting items extracted from: Luo X, Estill J, Chen Y (2023) The use of ChatGPT in medical research: do we need a reporting guideline? Int J Surg 109(12):3750-3751.
3.1 Use
Description: Did you use ChatGPT or similar software/tools to assist in writing the manuscript?
Explanation: If ChatGPT or similar tools were used during the writing of a research paper, it is suggested to report the details to enhance the transparency of the study.
Comment: This item emphasizes the use of the tool whatever the role, the section, or the requirement for reporting in detail. This item will be rated as "yes" as long as relevant documents mention this issue.
Example 1:
"At submission, authors must disclose whether they used AI or AI-assisted technologies in the preparation of the manuscript. Authors who use such technology must clearly describe, in both the cover letter and the manuscript, how AI or AI-assisted technologies (LARGE LANGUAGE MODELs, chatbots, such as ChatGPT or Bard, and image creators) were used in the study and/or manuscript preparation. Authors should be transparent when AI-assisted technologies are used and provide information about their use."
(extracted from website of *Radiology*; https://pubs.rsna.org/page/policies#llm).

Example 2:
"At submission, authors must disclose whether they used AI or AI-assisted technologies in the preparation of the manuscript. Authors who use such technology must clearly describe, in both the cover letter and the manuscript, how AI or AI-assisted technologies (LLMs, chatbots such as ChatGPT or Bard, and image creators) were used in the study and/or manuscript preparation. Authors should be transparent when AI-assisted technologies are used and provide information about their use. Authors may provide this information in the Materials and Methods section, the Acknowledgments section, or a relevant section of the manuscript (eg, figure legends for AI-generated figures). Authors should include specific details, such as the name and version of the AI tool, date of access, and name of the manufacturer or creator.
(extracted from Editorial of *Radiology*, Moy L. Guidelines for use of large language models by authors, reviewers, and editors: considerations for imaging journals. Radiology. 2023;309(1):e239024. https://doi.org/10.1148/radiol.239024).
Example 3:
"Authors are fully responsible for correctly labelling and disclosing which parts of their work has been created by or in assistance of AI"
(extracted from website of *European Radiology*; https://www.european-radiology.org/publication-ethics/#guidelines).
Example 4:
"Authors must disclose at submission whether they used AI or AI-assisted technologies in their work."
(extracted from Editorial of ESR Journals, Hamm B, Marti-Bonmati L, Sardanelli F. ESR Journals editors' joint statement on Guidelines for the Use of Large Language Models by Authors, Reviewers, and Editors. Eur Radiol 2024. https://doi/org/10.1007/s00330-023-10511-8).
Example 5:
"However, if such tools are used, the authors must report their use transparently. The report should include specific details, such as the name and version of the AI tool, date of access, name of the manufacturer/creator, and a comprehensive explanation of the use in the study conduct and manuscript writing. Authors may provide this information in a relevant section of the manuscript (e.g., figure legends for AI-generated figures) or collectively in the Acknowledgments section."
(extracted from Editorial of *Korean Journal of Radiology*, Park SH. Use of generative artificial intelligence, including large language models such as ChatGPT, in scientific publications: policies of KJR and prominent authorities. Korean J Radiol 2023;24(8):715-718. https://doi.org/10.3348/kjr.2023.0643).

3.2 Tool
Description: Please specify the AI tool and its version that was used in writing the manuscript.
Explanation: Authors should disclose in the relevant sections of the paper the name (e.g., ChatGPT, Claud, Bard, or NewBing) and the version (e.g., ChatGPT 3.5 or 4.0) of the AI tool(s) that were used.
Comment: The document should describe in detail to require the author to report the name, version, access data, etc. of the tool. Only requiring the author to report "what tool" is not enough. On the other hand, declaration requirements such as"during the preparation of this paper, the author(s) used [NAME TOOL / SERVICE] to create [REASON]. After using this tool/service, the author(s) have reviewed and edited the content as required and take full responsibility for the content of the publication" can be marked as "yes" for both 3.2 Tool and 3.4 Role items.
Example 1:
"Authors may provide this information in the Materials and Methods section or in the Acknowledgments section or a relevant section of the manuscript (e.g., figure legends for AI-generated figures), Authors should include specific details, such as the name and version of the AI tool, date of access, name of the manufacturer/creator."
(extracted from website of *Radiology*; https://pubs.rsna.org/page/policies#llm).
Example 2:
"Authors should include specific details, such as the name and version of the AI tool, date of access, and name of the manufacturer or creator. Authors should include specific details, such as the name of the language model or tool, version number, and manufacturer."
(extracted from Editorial of *Radiology*, Moy L. Guidelines for use of large language models by authors, reviewers, and editors: considerations for imaging journals. Radiology. 2023;309(1):e239024. https://doi.org/10.1148/radiol.239024).
Example 3:
"AI-tools used for writing and content editing must be disclosed in the acknowledgements. AI-tools used to generate results must be described in detail in the methods section"
(extracted from website of *European Radiology*; https://www.european-radiology.org/publication-ethics/#guidelines).
Example 4:
"In all cases of use of AI-assisted technologies, authors should include specific details, such as the name and version of the AI tool, date of access, and name of the manufacturer/creator."
(extracted from Editorial of ESR Journals, Hamm B, Marti-Bonmati L, Sardanelli F. ESR Journals editors' joint statement on Guidelines for the Use of Large Language Models by Authors, Reviewers, and Editors. Eur Radiol 2024. https://doi/org/10.1007/s00330-023-10511-8).

Example 5:
“However, if such tools are used, the authors must report their use transparently. The report should include specific details, such as the name and version of the AI tool, date of access, name of the manufacturer/creator, and a comprehensive explanation of the use in the study conduct and manuscript writing. Authors may provide this information in a relevant section of the manuscript (e.g., figure legends for AI-generated figures) or collectively in the Acknowledgments section.”
(extracted from Editorial of *Korean Journal of Radiology*, Park SH. Use of generative artificial intelligence, including large language models such as ChatGPT, in scientific publications: policies of KJR and prominent authorities. Korean J Radiol 2023;24(8):715-718. https://doi.org/10.3348/kjr.2023.0643).

3.3 Section
Description: Please report the specific section of the manuscript where ChatGPT or similar tools were used.
Explanation: Authors should explicitly report in the article for which paragraphs or sections ChatGPT (or other similar tool) was used, to assist readers in better understanding and assessing the content and value of the paper.
Comment: The document should require the author to report the exact paragraph or section that used LLMs, instead of just ask the author to report “how to use” it.
Example 1:
“Authors are fully responsible for correctly labelling and disclosing which parts of their work has been created by or in assistance of AI”
(extracted from website of *European Radiology*; https://www.european-radiology.org/publication-ethics/#guidelines).
Example 2:
“Any content created by AI or AI-assisted tools must be labelled”
(extracted from Editorial of ESR Journals, Hamm B, Marti-Bonmati L, Sardanelli F. ESR Journals editors' joint statement on Guidelines for the Use of Large Language Models by Authors, Reviewers, and Editors. Eur Radiol 2024. https://doi/org/10.1007/s00330-023-10511-8).

3.4 Role
Description: Please describe the role of ChatGPT in assisting the manuscript writing process.
Explanation: In addition to reporting which paragraphs were generated with the assistance of ChatGPT, authors should specify the exact role of ChatGPT in the process. This may include, for example, language refinement, outlining ideas, or generating content.
Comment: The document should require the author to report what he exact role of the tool in the process. The general descriptions such as “how the AI tool are used”, “disclose the use of AI and AI-assisted technologies in the writing process” are not enough. On the other hand, declaration requirements such as“during the preparation of this paper, the author(s) used [NAME TOOL / SERVICE] to create [REASON]. After using this tool/service, the author(s) have reviewed and edited the content as required and take full responsibility for the content of the publication” can be marked as “yes” for both 3.2 Tool and 3.4 Role items.
Example 1:
“Authors may use LARGE LANGUAGE MODELs to assist with medical writing and for content editing to effectively communicate their work. These tasks include assistance with medical writing, grammar, language and reporting standards. Authors must transparently report how they used such tools in the writing or editing of their submitted work, both in the cover letter and in the Methods section or the Acknowledgment section.”
(extracted from website of *Radiology*; https://pubs.rsna.org/page/policies#llm).
Example 2:
“Authors may use LLMs to assist with medical writing and for content editing to effectively communicate their work. These tasks include assistance with medical writing, grammar, language, and reporting standards. Authors must transparently re- port how they used such tools in the writing or editing of their submitted work, both in the cover letter and in the Methods section or the Acknowledgment section. The submission and publication of content and/ or images created by AI, language models, machine learning, or similar technologies is discouraged, un- less part of formal research design or methods, and is not permitted without clear description of the content that was created and the name of the model or tool, version and extension numbers, and manufacturer.”
(extracted from Editorial of *Radiology*, Moy L. Guidelines for use of large language models by authors, reviewers, and editors: considerations for imaging journals. Radiology. 2023;309(1):e239024. https://doi.org/10.1148/radiol.239024).

Example 3:
"Authors should be transparent when AI-assisted technologies are used and provide information about their use. If the tools were part of carrying out the research and to generate results, authors must provide this information in the Materials and Methods section or in the relevant section of the manuscript (e.g., figure legends for AI-generated figures). Authors may use LLMs to assist with medical writing and for content editing to effectively communicate their work. These tasks include assistance with medical writing, grammar, language, and reporting standards. Authors must transparently report how they used such tools in the writing or editing of their submitted work in the Acknowledgment section."
(extracted from Editorial of ESR Journals, Hamm B, Marti-Bonmati L, Sardanelli F. ESR Journals editors' joint statement on Guidelines for the Use of Large Language Models by Authors, Reviewers, and Editors. Eur Radiol 2024. https://doi/org/10.1007/s00330-023-10511-8).
Example 4:
"KJR discourages the use of generative AI tools for
the primary purpose of creating any types of content for scientific manuscripts except for studies mentioned in point 5 below. The use of LARGE LANGUAGE MODELs or other AI tools to enhance the linguistic quality of a submission is considered acceptable. This includes improving grammatical accuracy, rectifying
typographical errors, enhancing formatting, ensuring clarity, etc. Such applications can be particularly beneficial for non-native English speakers and do not require specific disclosure."
(extracted from Editorial of *Korean Journal of Radiology*, Park SH. Use of generative artificial intelligence, including large language models such as ChatGPT, in scientific publications: policies of KJR and prominent authorities. Korean J Radiol 2023;24(8):715-718. https://doi.org/10.3348/kjr.2023.0643).

3.5 Verification
Description: Please report whether the content generated by ChatGPT was verified and/or modified. If not, please explain the reason.
Explanation: Content directly generated by ChatGPT may contain false or exaggerated information, so it is recommended to manually proofread or verify the generated content to ensure its accuracy and reliability, and revise it if necessary. For example, if ChatGPT was used solely for language refinement, further modifications may be necessary. If no verification was performed, the reason should be clearly stated.
Comment: This item emphasizes the verification performed by human. Declarations such as "Authors are fully responsible for the content of their manuscript, even those parts produced by an AI tool, and are thus liable for any breach of publication ethics" does not fulfill this item since it emphasizes the "responsibility". On the hand, a declaration "After using this tool/service, the author(s) have reviewed and edited the content as required and take full responsibility for the content of the publication" can be marked as "yes". This item is different from the requirements of 3.6 Influence, since this emphasizes the "correction by human".
Example 1:
"All authors are responsible for any submitted material that includes AI-assisted technologies. AI-assisted technologies cannot distinguish between true and false information. Therefore, authors should carefully review and edit the results of AI-assisted content, because AI can generate authoritative-sounding output that can be biased, incomplete, or partially or completely incorrect. Authors should be able to assert that there is no plagiarism in their paper, including in text and images produced by AI. Humans must ensure appropriate attribution to all quoted material, including full citations. Authors should acknowledge all sources (including material produced by the chatbot). Authorship attribution requires accountability for the submitted work. Further, authors are responsible for any text generated by a chatbot in their manuscript (including the accuracy of what is presented and the absence of plagiarism) and for acknowledging all sources (including material produced by the chatbot) and ensuring the accuracy and completeness of citations. ICMJE updated the 'Manuscript Preparation and Submission' section to state that 'referencing AI-generated material as the primary source is not acceptable'."
(extracted from website of *Radiology*; https://pubs.rsna.org/page/policies#llm).
Example 2:
"All authors are responsible for any submitted material that includes AI-assisted technologies. AI-assisted technologies cannot distinguish between true and false information. Therefore, authors should carefully review and edit the results of AI-assisted con- tent, because AI can generate authoritative-sounding output that can be biased, incomplete, or partially or completely incorrect. Authors should be able to assert that there is no plagiarism in their paper, including in text and im- ages produced by AI. Humans must ensure appropriate attribution to all quoted material, including full cita- tions (1). Authors should acknowledge all sources (including material produced by the chatbot). Authorship attribution requires accountability for the submitted work. Further, authors are responsible for any text generated by a chatbot in their manuscript (including the accuracy of what is presented and the absence of plagiarism) and for acknowledging all sources (including material produced by the chatbot) and ensuring the accuracy and completeness of citations (2). ICMJE up- dated the "Manuscript Preparation and Submission" section to state that "Referencing AI-generated material as the primary source is not acceptable""
(extracted from Editorial of *Radiology*, Moy L. Guidelines for use of large language models by authors, reviewers, and editors: considerations for imaging journals. Radiology. 2023;309(1):e239024. https://doi.org/10.1148/radiol.239024).

Example 3:
"All authors are fully responsible for any submitted material that includes AI-assisted technologies
AI-assisted technologies cannot distinguish between true and false information. Humans, i.e., the authors, are and remain fully responsible for the submitted manuscript. Authors should carefully review and edit the results of AI-assisted content, because AI can generate authoritative-sounding output that can be biased, incomplete, or partially or completely incorrect. Authors should be able to assert that there is no plagiarism in their paper, including in text and images produced by AI Humans must ensure appropriate attribution to all quoted material, including full citations [1]. Authors should acknowledge all sources (including material produced by AI-assisted tools). Authorship attribution requires accountability for the submitted work. Further, authors are responsible for any text generated by an AI-assisted tool in their manuscript (including the accuracy of what is presented and the absence of plagiarism) and for acknowledging all sources (including mate- rial produced by the AI-assisted tool) and ensuring the accuracy and completeness of citations. AI-generated material cannot be referenced as primary source."
(extracted from Editorial of ESR Journals, Hamm B, Marti-Bonmati L, Sardanelli F. ESR Journals editors' joint statement on Guidelines for the Use of Large Language Models by Authors, Reviewers, and Editors. Eur Radiol 2024. https://doi/org/10.1007/s00330-023-10511-8).

3.6 Influence
Description: Please report whether and how the ChatGPT-generated content can be expected to have influenced the overall conclusions and accuracy of the research.
Explanation: Authors should report whether and how the content generated by ChatGPT may have influenced the results. If ChatGPT was used for language editing correctly, it generally should not affect the content. However, if ChatGPT is used to directly generate content, it may also have an impact on the results.
Comment: The document should require the author to report, or to consider the potential influence of LLMs, i. e., whether and how they potentially influence on the results of content. This item is different from the requirements of 3.5 Verification, since this emphasizes the "impact of LLMs".
Example:
"However, it is important to note that all language models have limitations and are unable to replicate human creative and critical thinking. Human intervention with these tools is essential to ensure that content presented is accurate and appropriate to the reader. Sage therefore requires authors to be aware of the limitations of language models and to consider these in any use of LLMs in their submissions:
Objectivity: Previously published content that contains racist, sexist or other biases can be present in LLM-generated text, and minority viewpoints may not be represented. Use of LLMs has the potential to perpetuate these biases because the information is decontextualized and harder to detect.
Accuracy: LLMs can 'hallucinate' i. e. generate false content, especially when used outside of their domain or when dealing with complex or ambiguous topics. They can generate content that is linguistically but not scientifically plausible, they can get facts wrong, and they have been shown to generate citations that don't exist. Some LLMs are only trained on content published before a particular date and therefore present an incomplete picture.
Contextual understanding: LLMs cannot apply human understanding to the context of a piece of text, especially when dealing with idiomatic expressions, sarcasm, humour, or metaphorical language. This can lead to errors or misinterpretations in the generated content.
Training data: LLMs require a large amount of high-quality training data to achieve optimal performance. However, in some domains or languages, such data may not be readily available, limiting the usefulness of the model."
(extracted from policy of SAGE, https://us.sagepub.com/en-us/nam/chatgpt-and-generative-ai).

Supplementary Note S3 Reporting of the use of large language models in current study

Six potential reporting items for authors

Six potential reporting items extracted from: Luo X, Estill J, Chen Y (2023) The use of ChatGPT in medical research: do we need a reporting guideline? Int J Surg 109(12):3750-3751.

1. Use

Description: Did you use ChatGPT or similar software/tools to assist in writing the manuscript?
Explanation: If ChatGPT or similar tools were used during the writing of a research paper, it is suggested to report the details to enhance the transparency of the study.
Reporting: Yes, we used two LLMs. We asked them whether there is any journal explicitly stating that they do not allow authorship to large language models.

2. Tool

Description: Please specify the AI tool and its version that was used in writing the manuscript.
Explanation: Authors should disclose in the relevant sections of the paper the name (e.g., ChatGPT, Claud, Bard, or NewBing) and the version (e.g., ChatGPT 3.5 or 4.0) of the AI tool(s) that were used.
Reporting: We used the two LLMs: (1) ChatGPT (ChatGPT-3.5-Turbo, OpenAI, https://openai.com/chatgpt) and (2) ERNIE Bot (ERNIE V2.5.2, Baidu, https://yiyan.baidu.com), respectively, performed at 14:00 to 14:30 (UTC+8) on January 13, 2024.

3. Section

Description: Please report the specific section of the manuscript where ChatGPT or similar tools were used.
Explanation: Authors should explicitly report in the article for which paragraphs or sections ChatGPT (or other similar tool) was used, to assist readers in better understanding and assessing the content and value of the paper.
Reporting: We used the two LLMs to generate Figure 1. Figure 1 is the screen captures of conversations with ChatGPT and ERNIE Bot. We asked the LLMs: “Do you know of any radiological journal explicitly stating that they do not allow authorship to large language models such as ChatGPT?”, or “Do you know of any radiological journal explicitly stating that they do not allow authorship to large language models such as DRNIE Bot?”, respectively. The other sections of our manuscript were written by authors themselves without assistance from LLMs. The following is the generated screen captures.

It is interesting to note that both large language models responded using plausible-sounding and grammatically correct sentences. They did not know the policy on the authorship of large language models of any radiological journals (red line). However, they both suggest in the conversation that to check the policies of the specific journal (blue line). The ERNIE Bot even pointed out that the journals and academic institutions are currently grappling with how to handle the increasing role of artificial intelligence in research and publication – just like our study (green line).

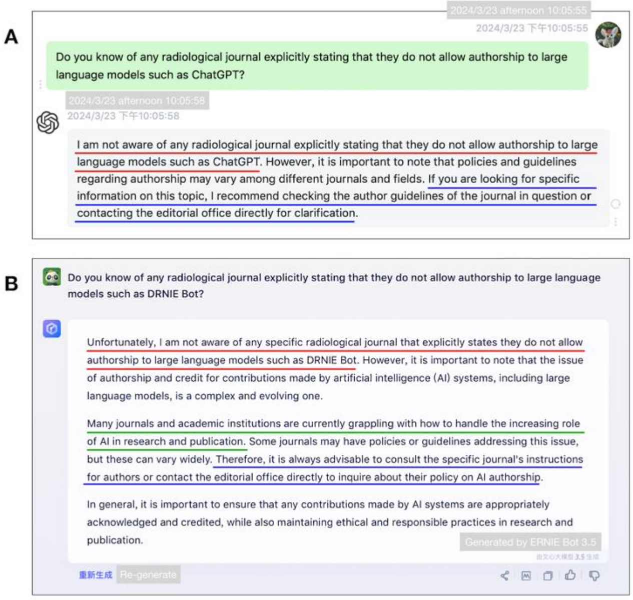

3.4 Role
Description: Please describe the role of ChatGPT in assisting the manuscript writing process.
Explanation: In addition to reporting which paragraphs were generated with the assistance of ChatGPT, authors should specify the exact role of ChatGPT in the process. This may include, for example, language refinement, outlining ideas, or generating content.
Reporting: We asked them whether there is any journal explicitly stating that they do not allow authorship to LLMs, in order to show the potential disadvantage of LLMs. They were not be used for other purpose.

3.5 Verification
Description: Please report whether the content generated by ChatGPT was verified and/or modified. If not, please explain the reason.
Explanation: Content directly generated by ChatGPT may contain false or exaggerated information, so it is recommended to manually proofread or verify the generated content to ensure its accuracy and reliability, and revise it if necessary. For example, if ChatGPT was used solely for language refinement, further modifications may be necessary. If no verification was performed, the reason should be clearly stated.
Reporting: After using this tool/service, the authors reviewed the content but did not edit it as needed. We did not edit it since we would like to show the potential disadvantage of LLMs. The authors take full responsibility for the content of the publication.

3.6 Influence
Description: Please report whether and how the ChatGPT-generated content can be expected to have influenced the overall conclusions and accuracy of the research.
Explanation: Authors should report whether and how the content generated by ChatGPT may have influenced the results. If ChatGPT was used for language editing correctly, it generally should not affect the content. However, if ChatGPT is used to directly generate content, it may also have an impact on the results.
Reporting: We have discussed the potential disadvantages of LLMs in the legend of Figure 1: “These two screen captures showed conversations with (A) ChatGPT (ChatGPT-3.5-Turbo, OpenAI, https://openai.com/chatgpt) and (B) ERNIE Bot (ERNIE V2.5.2, Baidu, https://yiyan.baidu.com), respectively, performed at 14:00 to 14:30 (UTC+8) on January 13, 2024. Both large language models responded using plausible-sounding and grammatically correct sentences. However, the ChatGPT did not know the policy on the authorship of large language models (red lines), since ChatGPT was not trained with data after September 2021. On the other hand, the ERNIE Bot gave an example of the Journal of American Medical Association declaring that the large language model “does not constitute authorship” (blue lines). Nevertheless, the so-called direct link to the author guidelines (https://jamanetwork.com/journals/jama/author-instructions) was not available (red lines).”

Supplementary Note S4 Representative examples for the presence of policies on the use of large language models in radiological journals

Example 1-1 Website guideline + Editorial
Journal:
RSNA Journals (*Radiology, RadioGraphic, Radiology Artificial Intelligence, Radiology Cardiovascular Imaging, Radiology Imaging Cancer*)
Website guideline:
https://pubs.rsna.org/page/radiology/author-instructions
Editorial:
Moy L. Guidelines for use of large language models by authors, reviewers, and editors: considerations for imaging journals. Radiology. 2023;309(1):e239024. https://doi.org/10.1148/radiol.239024
Relevant content (for authors and reviewers) on the website:

1. AI or AI-assisted technologies do not qualify as authors and must not be listed as authors or co-authors. Nonhuman AI, LLMs, chatbots, machine learning, or similar generative AI technologies do not meet the four ICMJE criteria for authorship. These qualifications were developed to guarantee that all authors accept full responsibility and stand for the integrity of the entire work. Accordingly, only humans can be authors. AI-assisted technologies should be reported in the article as methodological devices used in the completion of the work, but not included as authors.
2. At submission, authors must disclose whether they used AI or AI-assisted technologies in the preparation of the manuscript. Authors who use such technology must clearly describe, in both the cover letter and the manuscript, how AI or AI-assisted technologies (LLMs, chatbots, such as ChatGPT or Bard, and image creators) were used in the study and/or manuscript preparation. Authors should be transparent when AI-assisted technologies are used and provide information about their use. Authors may provide this information in the Materials and Methods section or in the Acknowledgments section or a relevant section of the manuscript (e.g., figure legends for AI-generated figures), Authors should include specific details, such as the name and version of the AI tool, date of access, name of the manufacturer/creator.
3. Authors may use LLMs to assist with medical writing and for content editing to effectively communicate their work. These tasks include assistance with medical writing, grammar, language and reporting standards. Authors must transparently report how they used such tools in the writing or editing of their submitted work, both in the cover letter and in the Methods section or the Acknowledgment section. Authors should include specific details, such as the name of the language model or tool, version number, and manufacturer.
4. All authors are responsible for any submitted material that includes AI-assisted technologies. AI-assisted technologies cannot distinguish between true and false information. Therefore, authors should carefully review and edit the results of AI-assisted content, because AI can generate authoritative-sounding output that can be biased, incomplete, or partially or completely incorrect.
5. Authors should be able to assert that there is no plagiarism in their paper, including in text and images produced by AI. Humans must ensure appropriate attribution to all quoted material, including full citations. Authors should acknowledge all sources (including material produced by the chatbot). Authorship attribution requires accountability for the submitted work. Further, authors are responsible for any text generated by a chatbot in their manuscript (including the accuracy of what is presented and the absence of plagiarism) and for acknowledging all sources (including material produced by the chatbot) and ensuring the accuracy and completeness of citations. ICMJE updated the 'Manuscript Preparation and Submission' section to state that 'referencing AI-generated material as the primary source is not acceptable'.
6. The submission and publication of content/images created by AI, language models, machine learning, or similar technologies is discouraged, unless part of formal research design or methods, and is not permitted without clear description of the content that was created and the name of the model or tool, version and extension numbers, and manufacturer. Authors must take responsibility for the integrity of the content generated by these models and tools. When generative AI itself is the focus of a study, the use of AI should be explicitly detailed.
7. The ICMJE and WAME guidelines states that reviewers should not upload the manuscript to software or other AI technologies where confidentiality cannot be assured. Reviewers are trusted and required to maintain confidentiality throughout the manuscript review process.

Note:
These journals have mentioned their documents in the submission guideline for authors. On the other hand, it is not expected that the real authors for these journals will try to search the relevant document on the journals' policies on the use of LLMs if it is not mentioned in the submission guideline for author. Therefore, we believe it is necessary to emphasize the relevant document in the submission guideline for author if it is published.

Example 1-2 Website guideline + Editorial
Journal:
ESR Journals (*European Radiology, Insights into Imaging, European Radiology Experimental*)
Website guideline:
https://www.european-radiology.org/publication-ethics/; https://www.i3-journal.org/publication-ethics/; https://www.er-x.org/for-authors/
Editorial:
Hamm B, Marti-Bonmati L, Sardanelli F. ESR Journals editors' joint statement on Guidelines for the Use of Large Language Models by Authors, Reviewers, and Editors. Eur Radiol. 2024. https://doi.org/10.1007/s00330-023-10511-8
Relevant content (for authors, reviewers, and editors) on the website:
Guidelines for the use of large language models by authors, reviewers, and editors
- AI or AI-assisted tools do not qualify as authors, only humans do.
- Authors are fully responsible for the entire content of their work.
- Authors are fully responsible for correctly labelling and disclosing which parts of their work has been created by or in assistance of AI:
  - AI-tools used to generate results must be described in detail in the methods section.
  - AI-tools used for writing and content editing must be disclosed in the acknowledgements.
- Reviewers and editors are obliged to confidentiality and should not upload manuscripts to software or AI-assisted tools where confidentiality cannot be assured.

Note:
These journals have mentioned their documents in the submission guideline for authors. On the other hand, it is not expected that the real authors for these journals will try to search the relevant document on the journals' policies on the use of LLMs if it is not mentioned in the submission guideline for author. Therefore, we believe it is necessary to emphasize the relevant document in the submission guideline for author if it is published.

Example 1-3 Website guideline + Editorial
Website guideline:
https://www.kjronline.org/index.php?body=Instruction
Editorial:
(1) Park SH. Authorship policy of the *Korean Journal of Radiology* regarding artificial intelligence large language models such as ChatGTP. Korean J Radiol 2023;24(3):171-172. https://doi.org/10.3348/kjr.2023.0112
(2) Park SH. Use of generative artificial intelligence, including large language models such as ChatGPT, in scientific publications: policies of KJR and prominent authorities. Korean J Radiol 2023;24(8):715-718. https://doi.org/10.3348/kjr.2023.0643
Relevant content (for authors and reviewers) on the website:
ETHICS AND AUTHORSHIP IN THE USE OF GENERATIVE ARTIFICIAL INTELLIGENCE (AI)
- Authorship assignment to AI is prohibited.
- Authors who employ generative AI tools are solely responsible for all content produced and submitted.
- *Korean Journal of Radiology* discourages the use of generative AI tools for the purpose of creating any types of content for scientific manuscripts. If such tools are used, the authors must report their use transparently.
- The use of AI tools to enhance the linguistic quality of a submission is considered acceptable and does not require specific disclosure.
- When generative AI itself is the focus of a study, the use of AI should be explicitly detailed in the Materials and Methods section.
- Reviewers are forbidden from using AI for the purpose of generating review comments.

Note:
This journal has mentioned their documents in the submission guideline for authors. On the other hand, it is not expected that the real authors for this journal will try to search the relevant document on the journal's policies on the use of LLMs if it is not mentioned in the submission guideline for author. Therefore, we believe it is necessary to emphasize the relevant document in the submission guideline for author if it is published.

Example 2-1 Website guideline
Journal:
American Journal of Roentgenology
Website guideline:
https://ajronline.org/authorguidelines
Relevant content (for authors):
Generative AI: Authors must disclose the use of large language models or other generative AI technologies in the manuscript’s preparation, including specific details regarding the relevant manuscript content, the particular technology applied (including name, version number, and manufacturer), and the manner in which the technology was used to create the content. Authors are responsible for any submitted materials created by generative AI technologies, including ensuring the accuracy of such material, the absence of plagiarism, and the appropriate attribution of sources.

Example 2-2 Website guideline
Journal:
Journal of Biomedical Optics
Website guideline:
https://www.spiedigitallibrary.org/journals/journal-of-biomedical-optics/author-guidelines
Relevant content (for authors):
Because authorship attribution requires accountability for the submitted work, SPIE does not allow ChatGPT and other Large Language Models (LLMs) to be listed as an author on a manuscript. If authors use AI tools when writing a manuscript, it should be disclosed along with all other tools used in the study in the Materials and Methods section of the paper. This section should describe which AI tool was used and how it was used. SPIE follows the guidance of COPE on this topic, which further advises that “Authors are fully responsible for the content of their manuscript, even those parts produced by an AI tool, and are thus liable for any breach of publication ethics.”

Example 2-3 Website guideline
Journal:
American Journal of Roentgenology
Website guideline:
https://ajronline.org/authorguidelines
Relevant content (for authors):
Generative AI: Authors must disclose the use of large language models or other generative AI technologies in the manuscript’s preparation, including specific details regarding the relevant manuscript content, the particular technology applied (including name, version number, and manufacturer), and the manner in which the technology was used to create the content. Authors are responsible for any submitted materials created by generative AI technologies, including ensuring the accuracy of such material, the absence of plagiarism, and the appropriate attribution of sources.

Example 2-4 Website guideline
Journal:
Current Medical Imaging
Website guideline:
https://www.eurekaselect.com/pages/author-guidelines
Relevant content (for authors):
AUTHORSHIP AND AI TOOLS
Bentham Science Publishers recognizes that authors use a variety of tools for preparing articles related to their scientific works, ranging from simple ones to very sophisticated ones.
According to the COPE (Committee on Publication Ethics) guidelines, "AI tools cannot meet the requirements for authorship as they cannot take responsibility for the submitted work. As non-legal entities, they cannot assert the presence or absence of conflicts of interest nor manage copyright and license agreements".
The pertinence of such tools may vary and evolve with public opinion, due to which the use of AI-powered language tools has led to a significant debate. These tools may generate useful results, but they can also lead to errors or misleading results; therefore, it is important to know which tools were used for evaluating and interpreting a particular scientific work.
Considering the above we require that:
The authors to report any significant use of such tools in their works, such as instruments and software along with text-to-text generative AI consistent with subject standards for methodology.
All co-authors should sign a declaration that they take full responsibility for all of its contents, regardless of how the contents were generated. Inappropriate language, plagiarized and biased contents, errors, mistakes, incorrect references, or misleading content generated by AI language tools and the relevant results reported in scientific works are the full and shared responsibility of all the authors, including co-authors.
AI language tools should not be listed as an author; instead, authors should follow clause (1) above.

Example 2-5 Website guideline
Journal:
Nuclear Medicine Communications
Website guideline:
https://edmgr.ovid.com/nmc/accounts/ifauth.htm
Relevant content (for authors):
Authors who use AI tools in the writing of a manuscript, production of images or graphical elements of the paper, or in the collection and analysis of data, must be transparent in disclosing in the Materials and Methods (or similar section) of the paper how the AI tool was used and which tool was used. Authors are fully responsible for the content of their manuscript, even those parts produced by an AI tool, and are thus liable for any breach of publication ethics.

Example 2-6 Website guideline
Journal:
Current Radiopharmaceuticals
Website guideline:
https://www.eurekaselect.com/journal/95/ifa
Relevant content (for authors):
AUTHORSHIP AND AI TOOLS
Bentham Science Publishers recognizes that authors use a variety of tools for preparing articles related to their scientific works, ranging from simple ones to very sophisticated ones.
According to the COPE (Committee on Publication Ethics) guidelines, "AI tools cannot meet the requirements for authorship as they cannot take responsibility for the submitted work. As non-legal entities, they cannot assert the presence or absence of conflicts of interest nor manage copyright and license agreements".
The pertinence of such tools may vary and evolve with public opinion, due to which the use of AI-powered language tools has led to a significant debate. These tools may generate useful results, but they can also lead to errors or misleading results; therefore, it is important to know which tools were used for evaluating and interpreting a particular scientific work.
Considering the above we require that:
The authors to report any significant use of such tools in their works, such as instruments and software along with text-to-text generative AI consistent with subject standards for methodology.
All co-authors should sign a declaration that they take full responsibility for all of its contents, regardless of how the contents were generated. Inappropriate language, plagiarized and biased contents, errors, mistakes, incorrect references, or misleading content generated by AI language tools and the relevant results reported in scientific works are the full and shared responsibility of all the authors, including co-authors.
AI language tools should not be listed as an author; instead, authors should follow clause (1) above.

Example 2-7 Website guideline
Journal:
Quantitative Imaging in Medicine and Surgery
Website guideline:
https://qims.amegroups.org/pages/view/guidelines-for-reviewers
Relevant content (for reviewers):
All unpublished manuscripts are confidential documents. The existence of a manuscript under review should not be revealed to anyone other than the peer reviewers and editorial staff. Peer reviewers are required to maintain confidentiality regarding the manuscripts they review and must not divulge any information about a specific manuscript or its content to any third party without prior permission from the journal’s editors, which may prohibit the uploading of the manuscript to software or other AI technologies where confidentiality cannot be assured. Reviewers should disclose to editors if and how AI technology is being used to facilitate their review and be aware that AI can generate authoritative-sounding output that can be incorrect, incomplete, or biased.

Example 3-1 Hyperlink to policies from the publisher
Journal:
Medical Image Analysis
Website guideline:
https://onlinelibrary.wiley.com/page/journal/20546750/homepage/forauthors.html
Relevant content (for authors):
Declaration of generative AI in scientific writing
The below guidance only refers to the writing process, and not to the use of AI tools to analyse and draw insights from data as part of the research process.
Where authors use generative artificial intelligence (AI) and AI-assisted technologies in the writing process, authors should only use these technologies to improve readability and language. Applying the technology should be done with human oversight and control, and authors should carefully review and edit the result, as AI can generate authoritative-

sounding output that can be incorrect, incomplete or biased. AI and AI-assisted technologies should not be listed as an author or co-author, or be cited as an author. Authorship implies responsibilities and tasks that can only be attributed to and performed by humans, as outlined in Elsevier's AI policy for authors.
Authors should disclose in their manuscript the use of AI and AI-assisted technologies in the writing process by following the instructions below. A statement will appear in the published work. Please note that authors are ultimately responsible and accountable for the contents of the work.
Policy from the publisher (Elsevier):
https://www.elsevier.com/about/policies-and-standards/publishing-ethics#Authors
Relevant content (for reviewers):
The use of generative AI and AI-assisted technologies in the journal editorial process
This policy has been triggered by the rise of generative AI and AI-assisted technologies* and aims to provide greater transparency and guidance to authors, editors and reviewers. Elsevier will monitor ongoing developments in this area closely and will adjust or refine the policy as appropriate. The following guidance is specifically for editors.
A submitted manuscript must be treated as a confidential document. Editors should not upload a submitted manuscript or any part of it into a generative AI tool as this may violate the authors' confidentiality and proprietary rights and, where the paper contains personally identifiable information, may breach data privacy rights.
This confidentiality requirement extends to all communication about the manuscript including any notification or decision letters as they may contain confidential information about the manuscript and/or the authors. For this reason, editors should not upload their letters into an AI tool, even if it is just for the purpose of improving language and readability.
Peer review is at the heart of the scientific ecosystem and Elsevier abides by the highest standards of integrity in this process. Managing the editorial evaluation of a scientific manuscript implies responsibilities that can only be attributed to humans. Generative AI or AI-assisted technologies should not be used by editors to assist in the evaluation or decision-making process of a manuscript as the critical thinking and original assessment needed for this work is outside of the scope of this technology and there is a risk that the technology will generate incorrect, incomplete or biased conclusions about the manuscript. The editor is responsible and accountable for the editorial process, the final decision and the communication thereof to the authors.
Elsevier's AI author policy states that *authors* are allowed to use generative AI and AI-assisted technologies in the writing process before submission, but only to improve the language and readability of their paper and with the appropriate disclosure, as per our instructions in Elsevier's Guide for Authors (opens in new tab/window). Editors can find such disclosure at the bottom of the paper in a separate section before the list of references. If an editor suspects that an author or a reviewer has violated our AI policies, they should inform the publisher.
Please note that Elsevier owns identity protected AI-assisted technologies which conform to the RELX Responsible AI Principles (opens in new tab/window), such as those used during the screening process to conduct completeness and plagiarism checks and identify suitable reviewers. These in-house or licensed technologies respect author confidentiality. Our programs are subject to rigorous evaluation of bias and are compliant with data privacy and data security requirements.
Elsevier embraces new AI-driven technologies that support reviewers and editors in the editorial process, and we continue to develop and adopt in-house or licensed technologies that respect authors', reviewers' and editors' confidentiality and data privacy rights.
** Generative AI is a type of artificial intelligence technology that can produce various types of content including text, imagery, audio and synthetic data. Examples include ChatGPT, NovelAI, Jasper AI, Rytr AI, DALL-E, etc.*
Relevant content (for authors):
The use of generative AI and AI-assisted technologies in scientific writing
This policy has been triggered by the rise of generative AI and AI-assisted technologies which are expected to increasingly be used by content creators. The policy aims to provide greater transparency and guidance to authors, readers, reviewers, editors and contributors. Elsevier will monitor this development and will adjust or refine this policy when appropriate. Please note the policy only refers to the writing process, and not to the use of AI tools to analyse and draw insights from data as part of the research process.
Where authors use generative AI and AI-assisted technologies in the writing process, these technologies should only be used to improve readability and language of the work. Applying the technology should be done with human oversight and control and authors should carefully review and edit the result, because AI can generate authoritative-sounding output that can be incorrect, incomplete or biased. The authors are ultimately responsible and accountable for the contents of the work.
Authors should disclose in their manuscript the use of AI and AI-assisted technologies and a statement will appear in the published work. Declaring the use of these technologies supports transparency and trust between authors, readers, reviewers, editors and contributors and facilitates compliance with the terms of use of the relevant tool or technology.
Authors should not list AI and AI-assisted technologies as an author or co-author, nor cite AI as an author. Authorship implies responsibilities and tasks that can only be attributed to and performed by humans. Each (co-) author is accountable for ensuring that questions related to the accuracy or integrity of any part of the work are appropriately investigated and resolved and authorship requires the ability to approve the final version of the work and agree to its submission. Authors are also responsible for ensuring that the work is original, that the stated authors qualify for

authorship, and the work does not infringe third party rights, and should familiarize themselves with our Ethics in Publishing policy before they submit.
The use of generative AI and AI-assisted tools in figures, images and artwork
We do not permit the use of Generative AI or AI-assisted tools to create or alter images in submitted manuscripts. This may include enhancing, obscuring, moving, removing, or introducing a specific feature within an image or figure. Adjustments of brightness, contrast, or color balance are acceptable if and as long as they do not obscure or eliminate any information present in the original. Image forensics tools or specialized software might be applied to submitted manuscripts to identify suspected image irregularities.
The only exception is if the use of AI or AI-assisted tools is part of the research design or research methods (such as in AI-assisted imaging approaches to generate or interpret the underlying research data, for example in the field of biomedical imaging). If this is done, such use must be described in a reproducible manner in the methods section. This should include an explanation of how the AI or AI-assisted tools were used in the image creation or alteration process, and the name of the model or tool, version and extension numbers, and manufacturer. Authors should adhere to the AI software's specific usage policies and ensure correct content attribution. Where applicable, authors could be asked to provide pre-AI-adjusted versions of images and/or the composite raw images used to create the final submitted versions, for editorial assessment.
The use of generative AI or AI-assisted tools in the production of artwork such as for graphical abstracts is not permitted. The use of generative AI in the production of cover art may in some cases be allowed, if the author obtains prior permission from the journal editor and publisher, can demonstrate that all necessary rights have been cleared for the use of the relevant material, and ensures that there is correct content attribution.
Note:
We did not mark all the Elsevier journals as "presence" of policy on the use of LLMs, since the description is not always in the submission guideline for author. It is not expected that the real authors for this journal will notice the publisher of these journals will try to find the policy on the use of LLMs if it is not mentioned in the submission guideline for author. However, the publisher's guideline does provide relevant information. Therefore, we believe it is necessary to emphasize the hyperlinks to the publisher's policies in the submission guideline for author if the journal has not its own policy website or document.

Example 3-2 Hyperlink to policies from the publisher
Journal:
Egyptian Journal of Radiology and Nuclear Medicine
Policy from the publisher (Springer Open):
https://www.springeropen.com/get-published/editorial-policies
Relevant content (for authors):
AI Authorship
Large Language Models (LLMs), such as ChatGPT, do not currently satisfy our authorship criteria. Notably an attribution of authorship carries with it accountability for the work, which cannot be effectively applied to LLMs. Use of an LLM should be properly documented in the Methods section (and if a Methods section is not available, in a suitable alternative part) of the manuscript.
Generative AI Images
The fast moving area of generative AI image creation has resulted in novel legal copyright and research integrity issues. As publishers, we strictly follow existing copyright law and best practices regarding publication ethics. While legal issues relating to AI-generated images and videos remain broadly unresolved, Springer Nature journals are unable to permit its use for publication.
Exceptions are images/art obtained from agencies that we have contractual relationships with that have created images in a legally acceptable manner. Other exceptions to this policy include images and video that are directly referenced in a piece that is specifically about AI and will be reviewed on a case-by-case basis.
As we expect things to develop rapidly in this field in the near future, we will review this policy regularly and adapt it if necessary.
Please note: Not all AI tools are generative. The use of non-generative machine learning tools to manipulate, combine or enhance existing images or figures should be disclosed in the relevant caption upon submission to allow a case-by-case review.
Note:
We did mark all the Springer Open journals (but not all the Springer Link journals) as "presence" of policy on the use of LLMs, since the description is always in the submission guideline for author. Although the journals did not provide their own policies, they do refer to the publisher's guideline does provide relevant information.

Example 3-3 Hyperlink to policies from the publisher
Journal:
Journal of Cardiovascular Magnetic Resonance
Policy from the publisher (BMC):
https://www.biomedcentral.com/getpublished/editorial-policies
Relevant content (for authors):
AI Authorship
Large Language Models (LLMs), such as ChatGPT, do not currently satisfy our authorship criteria. Notably an attribution of authorship carries with it accountability for the work, which cannot be effectively applied to LLMs. Use of an LLM should be properly documented in the Methods section (and if a Methods section is not available, in a suitable alternative part) of the manuscript.
Generative AI Images
The fast moving area of generative AI image creation has resulted in novel legal copyright and research integrity issues. As publishers, we strictly follow existing copyright law and best practices regarding publication ethics. While legal issues relating to AI-generated images and videos remain broadly unresolved, Springer Nature journals are unable to permit its use for publication.
Exceptions are images/art obtained from agencies that we have contractual relationships with that have created images in a legally acceptable manner. Other exceptions to this policy include images and video that are directly referenced in a piece that is specifically about AI and will be reviewed on a case-by-case basis.
As we expect things to develop rapidly in this field in the near future, we will review this policy regularly and adapt it if necessary.
Please note: Not all AI tools are generative. The use of non-generative machine learning tools to manipulate, combine or enhance existing images or figures should be disclosed in the relevant caption upon submission to allow a case-by-case review.
Note:
We did mark all the BMC journals (but not all the Springer Link journals) as "presence" of policy on the use of LLMs, since the description is always in the submission guideline for author. Although the journals did not provide their own policies, they do refer to the publisher's guideline does provide relevant information.

Example 3-4 Hyperlink to policies from the publisher
Journal:
Acta Radiologica Open
Website guideline:
https://journals.sagepub.com/author-instructions/ARR
Relevant content (for authors):
Acquisition of funding, collection of data, or general supervision of the research group alone does not constitute authorship, although all contributors who do not meet the criteria for authorship should be listed in the Acknowledgments section. Please refer to the International Committee of Medical Journal Editors (ICMJE) authorship guidelines for more information on authorship.
Please note that AI chatbots, for example ChatGPT, should not be listed as authors. For more information see the policy on Use of ChatGPT and generative AI tools.
Policy from the publisher (SAGE):
https://us.sagepub.com/en-us/nam/chatgpt-and-generative-ai
ChatGPT and Generative AI
Use of Large Language Models and generative AI tools in writing your submission
Sage recognises the value of large language models (LLMs) (e.g. ChatGPT) and generative AI as productivity tools that can help authors in preparing their article for submission; to generate initial ideas for a structure, for example, or when summarizing, paraphrasing, language polishing etc. However, it is important to note that all language models have limitations and are unable to replicate human creative and critical thinking. Human intervention with these tools is essential to ensure that content presented is accurate and appropriate to the reader. Sage therefore requires authors to be aware of the limitations of language models and to consider these in any use of LLMs in their submissions:
Objectivity: Previously published content that contains racist, sexist or other biases can be present in LLM-generated text, and minority viewpoints may not be represented. Use of LLMs has the potential to perpetuate these biases because the information is decontextualized and harder to detect.
Accuracy: LLMs can 'hallucinate' i.e. generate false content, especially when used outside of their domain or when dealing with complex or ambiguous topics. They can generate content that is linguistically but not scientifically plausible, they can get facts wrong, and they have been shown to generate citations that don't exist. Some LLMs are only trained on content published before a particular date and therefore present an incomplete picture.
Contextual understanding: LLMs cannot apply human understanding to the context of a piece of text, especially when dealing with idiomatic expressions, sarcasm, humour, or metaphorical language. This can lead to errors or misinterpretations in the generated content.

Training data: LLMs require a large amount of high-quality training data to achieve optimal performance. However, in some domains or languages, such data may not be readily available, limiting the usefulness of the model.
Guidance for authors
Authors are required to:
Clearly indicate the use of language models in the manuscript, including which model was used and for what purpose. Please use the methods or acknowledgements section, as appropriate.
Verify the accuracy, validity, and appropriateness of the content and any citations generated by language models and correct any errors or inconsistencies.
Provide a list of sources used to generate content and citations, including those generated by language models.
Double-check citations to ensure they are accurate, and are properly referenced.
Be conscious of the potential for plagiarism where the LLM may have reproduced substantial text from other sources. Check the original sources to be sure you are not plagiarising someone else's work.
Acknowledge the limitations of language models in the manuscript, including the potential for bias, errors, and gaps in knowledge.
Please note that AI bots such as ChatGPT should not be listed as an author on your submission.
We will take appropriate corrective action where we identify published articles with undisclosed use of such tools.
Authors should check the guidelines of the journal they are submitting to for any specific policies that may be in place on that journal.
General information on author responsibilities can be found on the Author Gateway.
Guidance for editors and reviewers
Editors and reviewers should evaluate the appropriateness of the use of LLMs and ensure that the generated content is accurate and valid.
Editors and Reviewers must uphold the confidentiality of the peer review process. Editors must not share information about submitted manuscripts or peer review reports with generative AI or LLMs such a ChatGPT. Reviewers must not use artificial intelligence tools to generate review reports, including but not limited to ChatGPT.
Further information
Please see the World Association of Medical Editors (WAME) recommendations on chat bots, ChatGPT and scholarly manuscripts and the Committee on Publication Ethics (COPE)'s position statement on Authorship and AI tools.
This policy may evolve further as we work with our publishing partners to understand how emerging technologies can help or hinder the process of preparing research for publication. Please check back to this page for the latest information.

Note:

We did mark all the SAGE journals as "presence" of policy on the use of LLMs, since the description is always in the submission guideline for author. Although the journals did not provide their own policies, they do refer to the publisher's guideline does provide relevant information.

Example 3-5 Hyperlink to policies from the publisher

Journal: *Sonography*

Website guideline:
https://onlinelibrary.wiley.com/page/journal/20546750/homepage/forauthors.html

Relevant content (for authors):

Artificial Intelligence-Generated Content
Please refer to Wiley's Best Practice Guidelines for details regarding AIGC ('Authorship' section). As a rule, AI and LLM entities do not qualify for authorship and the use of such tools should be fully declared during submission.

Policy from the publisher (Wiley):
https://authorservices.wiley.com/ethics-guidelines/index.html

Relevant content (for authors):

Artificial Intelligence Generated Content
Artificial Intelligence Generated Content (AIGC) tools—such as ChatGPT and others based on large language models (LLMs)—cannot be considered capable of initiating an original piece of research without direction by human authors. They also cannot be accountable for a published work or for research design, which is a generally held requirement of authorship (as discussed in the previous section), nor do they have legal standing or the ability to hold or assign copyright. Therefore—in accordance with COPE's position statement on AI tools—these tools cannot fulfill the role of, nor be listed as, an author of an article. If an author has used this kind of tool to develop any portion of a manuscript, its use must be described, transparently and in detail, in the Methods or Acknowledgements section. The author is fully responsible for the accuracy of any information provided by the tool and for correctly referencing any supporting work on which that information depends. Tools that are used to improve spelling, grammar, and general editing are not included in the scope of these guidelines. The final decision about whether use of an AIGC tool is appropriate or permissible in the circumstances of a submitted manuscript or a published article lies with the journal's editor or other party responsible for the publication's editorial policy.

Note:
We did not mark all the Wiley journals as "presence" of policy on the use of LLMs, since the description is not always in the submission guideline for author. It is not expected that the real authors for this journal will notice the publisher of these journals will try to find the policy on the use of LLMs if it is not mentioned in the submission guideline for author. However, the publisher's guideline does provide relevant information. Therefore, we believe it is necessary to emphasize the hyperlinks to the publisher's policies in the submission guideline for author if the journal has not its own policy website or document.

Example 3-6 Hyperlink to policies from the publisher
Journal:
Journal of Visual Communication in Medicine
Website guideline:
https://www.tandfonline.com/action/authorSubmission?show=instructions&journalCode=ijau20
Relevant content (for authors):
Defining authorship
It is the collective responsibility of all the individuals who have conducted the work to determine who should be listed as authors, and the order in which authors should be listed.
The journal editor will not decide on order of authorship and cannot arbitrate authorship disputes. Where unresolved disputes between the authors arise, the institution(s) where the work was performed will be asked to investigate.
Please read our guide to defining authorship. It includes details on:

- Corresponding authors
- Changes in authorship
- Assistance from scientific, medical, technical writers or translators
- Assistance with experiments and data analysis
- Acknowledgments
- Author name change policy

Policy from the publisher (Taylor & Francis):
https://authorservices.taylorandfrancis.com/editorial-policies/defining-authorship-research-paper/
Relevant content (for authors):
AI-based tools and technologies for content generation
Authors must be aware that using AI-based tools and technologies for article content generation, e. g. large language models (LLMs), generative AI, and chatbots (e.g. ChatGPT), is not in line with our authorship criteria.
All authors are wholly responsible for the originality, validity and integrity of the content of their submissions. Therefore, LLMs and other similar types of tools do not meet the criteria for authorship.
Note:
We did not mark this Taylor & Francis journal as "presence" of policy on the use of LLMs, since the description is not always in the submission guideline for author. It is not expected that the real authors for this journal will notice the publisher of these journals will try to find the policy on the use of LLMs if it is not mentioned in the submission guideline for author. However, the publisher's guideline does provide relevant information. Therefore, we believe it is necessary to emphasize the hyperlinks to the publisher's policies in the submission guideline for author if the journal has not its own policy website or document.
